# Elevated CO_2_ alters relative belowground carbon investment for nutrient acquisition in a mature temperate forest

**DOI:** 10.1073/pnas.2503595122

**Published:** 2025-07-15

**Authors:** Michaela K. Reay, Emma J. Sayer, Andrew Smith, Victoria Pastor, Angeliki Kourmouli, Miles Marshall, Robert T. Grzesik, Iwan Evans, Manon Rumeau, Kris Hart, Jiaojiao Ma, Richard J. Norby, A. Robert MacKenzie, R. Liz Hamilton, Iain P. Hartley, Sami Ullah

**Affiliations:** ^a^School of Geography, Earth and Environmental Science, University of Birmingham, Birmingham B15 2TT United Kingdom; ^b^Birmingham Institute of Forest Research, University of Birmingham, Birmingham B15 2TT, United Kingdom; ^c^Organic Geochemistry Unit, School of Chemistry, University of Bristol, Bristol BS8 1TS, United Kingdom; ^d^Lancaster Environment Centre, Lancaster University, Lancaster LA1 4YQ, United Kingdom; ^e^Institute of Botany, Ulm University, Ulm D-89081, Germany; ^f^School of Environmental and Natural Sciences, Bangor University, Bangor LL57 2UR, United Kingdom; ^g^Department of Biology, Biochemistry, and Natural Sciences, School of Technology and Experimental Sciences, Universitat Jaume I, Castelló de la Plana 12006, Spain; ^h^Environmental Sciences Division, Oak Ridge National Laboratory, Oak Ridge, TN 37830; ^i^Geography, Faculty of Science, Environment and Economy, University of Exeter, Exeter EX4 4RJ, United Kingdom

**Keywords:** free-air carbon enrichment, root exudation, ectomycorrhizal fungi, root morphology, relative response

## Abstract

Forests are potential carbon (C) sinks that partially offset anthropogenic carbon dioxide (CO_2_) emissions via enhanced C assimilation and productivity. However, the question remains whether mature trees will express sufficient plasticity in nutrient acquisition strategies to support enhanced growth under elevated CO_2_ (eCO_2_). Trees may sustain growth by investing C belowground to enhance nutrient acquisition, e.g., by increasing root absorptive surfaces for greater soil available resource exploration (a “do-it-yourself” strategy) or utilizing C exudation or mycorrhizal associations as priming mechanisms for nutrient acquisition (“outsourcing”). We show that 4 y of eCO_2_ (+140 ± 38 ppm; i.e., +35% above ambient) altered the relative belowground C investment strategies of mature oak (*Quercus robur* L.) in a 180-y-old temperate forest. Fine-root branching frequency increased 73% under eCO_2_. Specific root C exudation was enhanced under eCO_2_ (63%), particularly outside the peak growing season, and the exudate C to nitrogen (N) ratio was increased (28%). Ectomycorrhizal (ECM) biomass production increased during leaf fall (17%) while ECM turnover increased almost fourfold under eCO_2_. The exudate and root metabolome composition were considerably altered during the late growing season under eCO_2_. We find, therefore, that a broad suite of nutrient acquisition strategies are upregulated under eCO_2_, with dynamic shifting between different outsourcing and do-it-yourself elements at different times of the year. These belowground changes support the increase in net primary productivity observed in this forest, with implications for the role of mature temperate forests in the global carbon sink.

## Significance

Growth of forest trees in response to rising atmospheric carbon dioxide (CO_2_) will likely be constrained by nutrient availability, yet long-established forests are significantly underrepresented in such studies. Using the Birmingham Institute of Forest Research’s free-air carbon enrichment experiment during the fourth and fifth years of CO_2_ treatment, we report a greater overall investment in root/soil pathways for nutrient acquisition under elevated CO_2_. Dynamic seasonal changes demonstrated the importance of a multitude of nutrient acquisition mechanisms. These shifts in how carbon is allocated belowground have significant implications for the stability of soil organic matter, for the accuracy of Earth System models, and, ultimately, for the ability of mature forests to act as carbon sinks under future atmospheric CO_2_ levels.

The ability of forest ecosystems to help mitigate rising atmospheric carbon dioxide (CO_2_) concentrations is of global importance. Forests account for *c.* 82% of the terrestrial carbon (C) pool and contribute 0.72 ± 0.08 Pg C y^−1^ to the global carbon sink (1, 2). A large part of this C is stored in plant biomass, and increased net primary productivity (NPP) in forests in response to rising atmospheric CO_2_ (the CO_2_ fertilization effect) is thought to have played a crucial role in limiting the rise in atmospheric CO_2_ from anthropogenic emissions (2). Increased C inputs from aboveground litter (3), root biomass turnover (4), and root C exudation (5) have been observed under elevated CO_2_ (eCO_2_) in ecosystem-level free-air CO_2_ enrichment (FACE) experiments in young forests. However, the ability of mature forests to sequester additional C depends on the availability of sufficient nutrients and phenotypic plasticity to support extra growth (6). Without an exogenous supply of nutrients, the growth response of mature forests to rising atmospheric CO_2_ is likely to be constrained by nutrient availability as the nutrients become sequestered in wood or soil organic matter (SOM) during succession (7). However, mature forests are massively underrepresented in studies of ecosystem responses to eCO_2_. Second-generation FACE experiments are now testing how nutrient availability might constrain the response of mature forests to eCO_2_. For example, in a mature Eucalyptus Forest in Australia, the lack of increased productivity under eCO_2_ was attributed to low phosphorus (P) availability (8). By contrast, at the Birmingham Institute of Forest Research (BIFoR) FACE site in a mature temperate oak (*Quercus robur*) forest (~180 y old) in the United Kingdom, NPP and foliar nitrogen (N) increased (9, 10), alongside decreased soil nitrate under eCO_2_ (11). Additional N required for enhanced growth under eCO_2_ at BIFoR FACE is thought to derive from consistent upregulation of soil N transformations with a higher and faster ammonium availability and turnover at the study site (12, 13), combined with a small increase in standing fine root biomass (14), indicating that changes in belowground processes could support biomass C sequestration in mature forests under eCO_2_.

Changes in the investment of C belowground could increase the acquisition of nutrients to support enhanced growth of mature forests under eCO_2_. Plants can increase resource acquisition under elevated nutrient demand conditions via three major mechanisms: changing root system morphology, increasing root exudation, and investing in mycorrhizal associations (15). Although greater root production has also been identified as a major mechanism for exploring available soil resource and increasing plant nutrient uptake in young forests under eCO_2_ (16), root systems in mature forests may have already explored a greater volume of soil resources, which may constrain additional root expansion (17, 18). Instead, C investment in greater specific root length (SRL) or root branching intensity could enhance nutrient uptake under eCO_2_. Increased SRL is strongly associated with nutrient acquisition (19), while high root branching intensity allows rapid proliferation of roots into nutrient-rich microsites of soil (20). Enhanced investment of C in root exudation, alongside changes in exudate composition, could increase nutrient availability under eCO_2_ (21) by stimulating soil microbial activity to mineralize N bound in SOM (22). Indeed, some FACE studies have observed increased root exudation in young trees (23). Finally, trees can invest C in mycorrhizal fungi to increase N and P acquisition for sustained growth under eCO_2_ (24), which is reflected in increased mycorrhizal colonization and biomass (25). For example, greater tree biomass in ectomycorrhizal (ECM)-dominated young temperate forests under eCO_2_ was attributed to increased C allocation to mycorrhizal fungi, which enhanced rhizomorph production (26) and N uptake (16). Thus, all three mechanisms (absorptive root surfaces, exudation, and mycorrhizal associations) are potentially important for maintaining enhanced tree growth under eCO_2_ as demand for tree nutrients increases.

The primary mechanism used by trees to acquire nutrients depends strongly on both species’ identity and environmental conditions, but very few studies have considered all three mechanisms concomitantly (15). With increasing nutrient demand under eCO_2_ and greater availability of C through CO_2_ fertilization, it is possible there may be changes in investment in nutrient acquisition strategies. According to the root economic space (RES) framework, investment in increased root branching and SRL represent “do-it-yourself” resource acquisition strategies. Mycorrhizal associations or priming of soil microbial activity through exudation of labile C represent “outsourcing” in organic-rich forest soils (27), with the latter associated with fast nutrient acquisition/conservation (15). As resource requirements in mature temperate ecosystems vary seasonally (28), it is conceivable that investment in resource acquisition mechanisms differs during periods of leaf expansion, biomass growth, or leaf senescence. Understanding the strategies by which trees in mature forests acquire nutrients is essential to underpin enhanced growth and biomass production under eCO_2_ and subsequent C sequestration in temperate forests.

Here, we quantified belowground C investment in nutrient acquisition mechanisms in a mature forest under eCO_2_ and assessed seasonal changes in resource acquisition strategies. At BIFoR FACE, a mature deciduous forest dominated by 180-y-old *Quercus robur* L. in central England, treatment arrays (~30 m diameter) have been exposed to +140 ± 38 ppm (target 150 ppm; 2017 to 2021) above aCO_2_ since spring 2017 (29). Under eCO_2_, the trees have increased area-based foliar N content and photosynthetic rates (10, 30), and upregulated soil N transformations, indicating that changes in belowground nutrient cycling processes support plant N uptake (12). These observations indicate greater investment in resource acquisition strategies by mature trees, however, the mechanisms by which nutrient acquisition is supported at BIFoR FACE under eCO_2_ remain unclear. We therefore quantified relative investment in potential nutrient acquisition mechanisms (root morphology, exudates, and ECM associations) for 1 y to test the following hypotheses:

H1. Seasonal patterns of investment in nutrient acquisition mechanisms will synchronize with tree growth periods, with greater root branching, exudation, or ECM during the main growing season.
H2. Trees growing under eCO_2_ will invest more C in nutrient acquisition, with greater SRL, root branching, root exudation, and/or ECM biomass compared to trees growing under ambient CO_2_ (aCO_2_) concentrations.
H3. There will be trade-offs in C investment among nutrient acquisition mechanisms, with trees under eCO_2_ favoring root morphological changes (a do-it-yourself strategy) and exudation (an outsourcing strategy) as fast-growth strategies over investment in ECM associations.

We measured root exudation, root morphology, and ECM biomass production every 3 mo after 4 y of eCO_2_ enrichment of the oak-dominated mature forest at BIFoR FACE to assess the potential of each mechanism to enhance nutrient acquisition during late growth (August) and leaf fall (November) in 2020, and subsequent bud burst (March) and the peak growing season (June) in 2021. The 2020 growing season had average woody growth (normalized dry matter increment (dmi) ~ 45 kg tree^−1^), with an eCO_2_ effect of 19%, while 2021 had higher woody growth (dmi ~ 60 kg tree^−1^) but a smaller eCO_2_ effect (6.9%) (9). To aid interpretation, we also characterized root exudate composition and ECM turnover during the main growth period. In doing so, we reveal how seasonal changes in the resource acquisition strategies of trees could sustain enhanced growth under eCO_2_.

## Results and Discussion

**Fig. 1.**
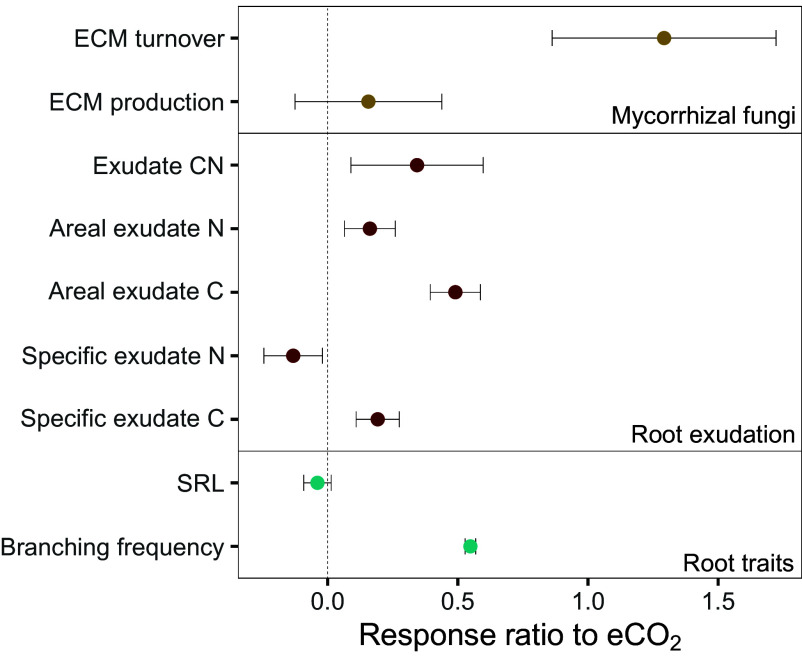
Annual RR showing the magnitude of changes in mycorrhizal, exudate, and root properties under eCO_2_ relative to ambient [RR = ln(R_e_/R_a_), where R_e_ is the response under eCO_2_, and R_a_ is the response under aCO_2_]; positive values indicate an increase, and negative values indicate a decrease under eCO_2_ relative to aCO_2_. Dots represent means for *n* = 3, across four timepoints with pseudoreplication of *n* = 18 for root exudation and root traits, and *n* = 15 for ECM. Error bars are ± variance (*v*). Response ratios refer to overall effects of eCO_2_ across the entire study period, and therefore do not indicate seasonal differences between treatments.

There is strong evidence of an annual CO_2_ treatment effect across nutrient acquisition strategies [relative response (RR) of eCO_2_ to aCO_2_ RR ≠ 0; Fig. 1], but with complex seasonal patterns and interrelationships which correspond to changes in the relative investment in do-it-yourself and outsourcing strategies.

### Changes in Root Morphology As a Mechanism for Nutrient Acquisition.

**Fig. 2.**
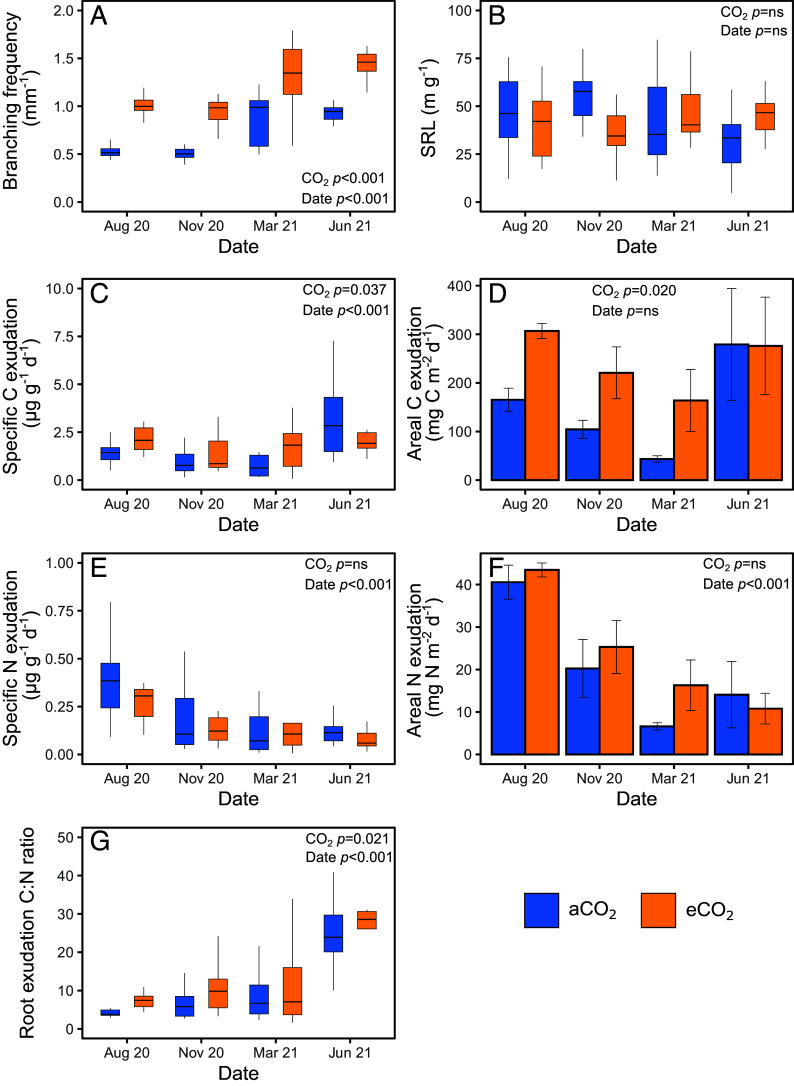
Fine root traits and exudation of mature oak trees under aCO_2_ and eCO_2_, showing (*A*) root branching frequency, (*B*) SRL, (*C*) specific root C exudation per unit root mass, (*D*) areal C exudation rate adjusted for total root biomass in the O horizon, (*E*) specific root N exudation per unit root mass, (*F*) areal N exudation rate adjusted for root biomass in the O horizon, and (*G*) exudate C:N ratio. For parts (*A*–*C*, *E*, and *G*), data are presented as box plots to reflect distribution of data, boxes denote the 25th and 75th percentiles and median lines are given for *n* = 18 pseudoreplicates, whiskers indicate values up to 1.5× the interquartile range. For parts (*D*) and (*F*), exudation rates on a mass basis were adjusted using root standing biomass on an array basis (*n* = 5), and values are mean ± SE (*n* = 3).

We first examined changes in root morphology (a do-it-yourself strategy) by measuring root branching and SRL, which are key root traits associated with plastic response to the environment and nutrient demands (19, 31). We found a consistent increase in root branching frequency under eCO_2_ compared to aCO_2_ across seasons (Fig. 2*A* and *SI Appendix*, Table S1, RR = 0.54), which suggests greater C investment in accessing and exploiting soil nutrient hotspots (32). Greater SRL under eCO_2_ during the main growth period in June 2021 is a result of increased root branching (Fig. 2*B*), when the requirement for additional nutrients and water is greatest, although there was no seasonal change for SRL unlike branching (*SI Appendix*, Table S1). At our study site, roots were most abundant in the organic horizon, where greater root branching could maximize the capture and retention of mobile nutrients, such as N, released during the mineralization of SOM (33). Increased root branching has been associated with greater aboveground growth under eCO_2_ in young forests (34). As the previously observed increase in woody biomass production is only a small proportion of mature tree total biomass, (9), changes in tree size and branching are considered relatively independent. Our results showing the highest branching intensity in spring and early summer suggest that this morphological adjustment boosts nutrient acquisition during the growing season to support increased NPP observed at BIFoR-FACE under eCO_2_ (9).

### Changes in Root Exudation and Exudate Composition under eCO_2_.

We next assess seasonal changes in net root exudation, expressed on a mass-specific basis, which were correlated with area-specific exudation rates (*SI Appendix*, Figs. S2 and S3). Net C exudation rates were higher (41 to 135%) under eCO_2_ compared to aCO_2_ (Fig. 2*C* and *SI Appendix*, Table S1), except in June 2021 (3.37 ± 0.76 μg g^−1^ d^−1^ under eCO_2_ and 4.06 ± 0.98 μg g^−1^ d^−1^ under aCO_2_). The overall increase in exudation under eCO_2_ supports our hypothesis that eCO_2_ increases belowground C investment in exudation, which may reflect excess available C from photosynthesis to invest. Higher exudation in the peak growing season at maximal photosynthesis will promote nutrient release in the soil by stimulating rhizosphere microbial activity (35) and coincides with higher rhizosphere activity and N supply under eCO_2_ observed at BIFoR FACE (13). C released during leaf senescence in the late growing season may also contribute to exudation, with the potential to prime microbes to support nutrient availability the following spring (35, 36). The largest difference in exudation under eCO_2_ was observed in March 2021 (RR = 0.85), which strongly points to an active control on C exudation to promote nutrient availability prior to budburst, in contrast to passive controls of C availability.

Seasonal changes in N exudation were also observed, however, there was no effect of eCO_2_ (*SI Appendix*, Table S1). N exudation was highest in the late growing season and then during leaf fall, with evidence of N conservation prior to bud bust and the peak growing season (Fig. 2*E*), coupled with higher C:N ratio (Fig. 2*G*). As net exudation measurement is a combination of release and reuptake of compounds in exudates, reuptake of exuded N across the diurnal cycle of exudate collection could enhance N use efficiency by trees under eCO_2_ and suggested tree N conservation. This is consistent with the tighter N cycling and upregulation of bulk and rhizosphere soil N transformations observed at our study site (12, 13). Although there was no effect of eCO_2_ on specific net N exudation rates, the areal amount of exuded N (exudation per m^2^ of forest) was nonetheless 18% higher under eCO_2_ than aCO_2_ (*P* = 0.049) due to larger standing fine root biomass in the eCO_2_ treatment in the period of the exudate experiment (RR = 0.16; *SI Appendix*, Fig. S4). The increase in areal N exudation will feedback on N availability in the rhizosphere and may indirectly act to prime SOM breakdown to support nutrient availability to both microbes and trees.

**Fig. 3.**
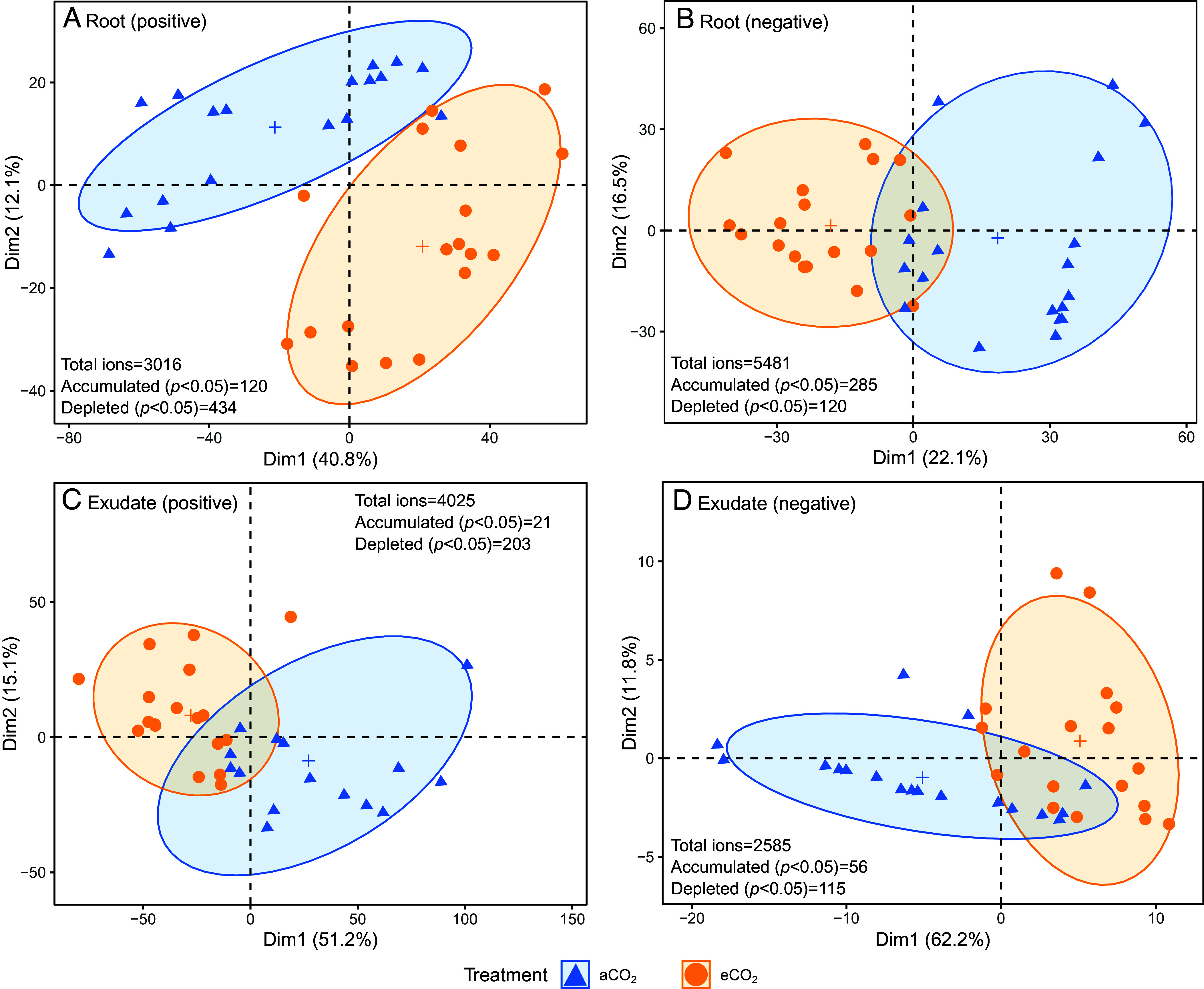
Ordination plots of untargeted metabolomics profiles of roots and exudates under aCO_2_ and eCO_2_, based on PCA, showing (*A*) root metabolome under positive ionization, (*B*) root metabolome under negative ionization, (*C*) exudate metabolome under positive ionization, and (*D*) exudate metabolome under negative ionization, analyzed via LC–MS. Each small, filled, symbol indicates an individual root/exudate, and the cross indicates the mean (*n* = 18). The ellipses indicate the 95% CI, and Dim1 and Dim2 indicate the variability explained by principal components 1 and 2, respectively. For each plot, the total number of features, and the number significantly (*P* < 0.05) accumulated or depleted under eCO_2_, determined via Kruskal–Wallis tests, are shown.

A higher exudate C:N ratio under eCO_2_ (Fig. 2*G*; RR = 0.34) supports our hypothesis of increased C investment in exudates to acquire N (21, 37) not clearly reflected in the root tissue C:N ratio (*SI Appendix*, Fig. S5). Trees adjust the proportions of compounds released as exudates into the soil in response to changes in their environment (37). To further explore shifts in root exudate composition under eCO_2_, we characterized exudate composition during the late growth period (August 2020) using untargeted metabolomics and principal component analysis (PCA) based on the abundance of ions, representative of the number of compounds exuded by roots. Although ions are not matched to chemical identity, changes in the abundance of ions provide a robust indication of changes in metabolite composition of exudates. This approach revealed significantly altered exudate composition under eCO_2_ (Fig. 3), driven by a small number of ions (6.0% of 6,610 ions present in total; *SI Appendix*, Table S2). Of the 395 individual ions that differed significantly (*SI Appendix*, Table S2), 77 were accumulated by 1.14 to 9.22-fold under eCO_2_ relative to aCO_2_ and 318 were depleted by −1.21 to −10.8-fold (*SI Appendix*, Figs. S8–S15). Previous targeted analyses of root exudation from *Pinus sylvestris* seedlings colonized by ECM showed increases in amino acids, carbohydrates, and organic acids under eCO_2_ (38). Given the lack of change in N exudation on a mass-specific basis, we suggest compounds with a higher C:N (or no N) are accumulated in exudates under eCO_2_ [e.g., carbohydrates, organic acids, lipids (38)], while compounds with a lower C:N ratio (e.g. amino acids, peptides, and proteins such as enzymes) may be unaffected or depleted (39).

We assessed the extent to which changes in the production of compounds under eCO_2_ might influence exudate composition by characterizing the root metabolome. We found 2,753 unique ions that were shared between exudates and roots, which likely consist of the core metabolome [e.g., amino acids, carbohydrates, organic acids, and lipids (40)]. Of the shared ions, only 14 were significantly accumulated or depleted under eCO_2_ (*SI Appendix*, Table S3), suggesting a core metabolome unaffected by eCO_2_ (40). Accumulation in roots, but depletion in exudates (*n* = 3) alongside depletion in both (*n* = 11), suggests modification in the relative release vs. production under eCO_2_ (39). Thus, a change in exudation rather than production likely drove the observed changes in exudation chemistry. Furthermore, only a small number of features in either the root or exudate metabolome differed significantly between aCO_2_ and eCO_2_ relative to the total metabolome (*n* = 402 and 77 accumulated, respectively, and *n* = 543 and 304 depleted, respectively; *SI Appendix*, Table S2). The combined changes in metabolomic features and exudate C:N ratio provide strong evidence of shifts in exudate composition under eCO_2_ outside of the core metabolome, which we hypothesize will support extended N acquisition via SOM mineralization. The idea that shifts in exudate composition could be related to N acquisition under eCO_2_ is further supported by rhizosphere suppression of nitrification relative to ammonification, suggesting biological nitrification inhibitors in the rhizosphere at the study site (13), which are regarded as an N-conservation mechanism in response to high tree N demands (41). Changes in exudate composition alter SOM formation and priming (36); thus, the observed changes in exudate composition have implications for carbon storage under eCO_2_.

### Increases in Mycorrhizal Biomass and Turnover.

**Fig. 4.**
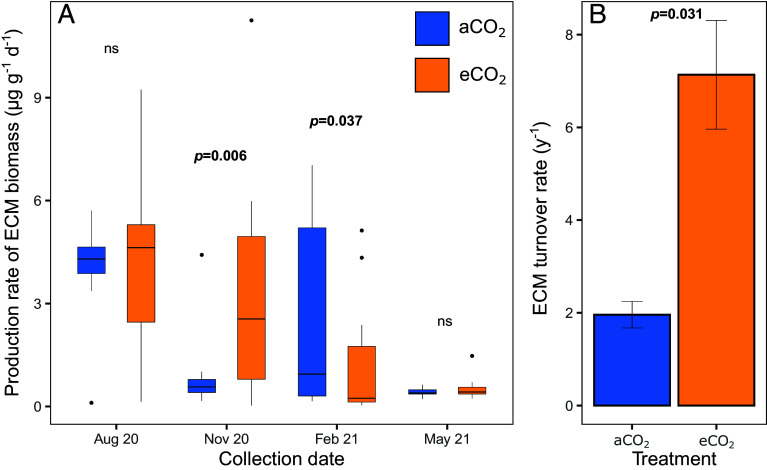
ECM biomass production rate (*A*) and turnover rate (*B*) under aCO_2_ and eCO_2_ in sand ingrowth bags installed for 3 mo (*A*) and 6 and 12 mo (*B*). Bold *P* values indicate a significant difference at the individual time point, determined via a Wilcoxon rank-sum test, and ns indicates not significant. Boxes denote the 25th and 75th percentiles and median lines are given for *n* = 18 pseudoreplicates, whiskers indicate values up to 1.5× the interquartile range, and dots indicate outliers.

Increased annual turnover of ECM likely arose from increased ECM production during leaf fall under eCO_2_. We assessed C investment in ECM associations (an outsourcing strategy), using the fungal biomarker ergosterol in seasonally harvested hyphal ingrowth bags to measure net ECM biomass production. We used staggered installation of ingrowth bags for 6 mo and 12 mo (i.e., May 2020 to May 2021) to determine annual ECM biomass turnover rates (42). The observed increased ECM production under eCO_2_ during the peak and particularly the late growing season was reflected in the 3.6-fold faster ECM turnover under eCO_2_ (7.13 ± 1.17 y^−1^) than under aCO_2_ (1.96 ± 0.56 y^−1^; *P* = 0.031; Fig. 4*B*). Although we cannot pinpoint seasonal changes in turnover, such a boost in ECM biomass turnover demonstrates substantial additional C investment into ECM associations. ECM turnover varies with stand age (43), with peak rates during stand development stages with the highest nutrient demand (44) which may explain previous lack of response in young trees (45, 46) while a clear increase was observed herein. In the mature forest in our study, we demonstrate increased annual turnover likely arose from increased ECM biomass production during specific growth periods under eCO_2_, which supports our hypothesis that substantial amounts of C are being invested in nutrient acquisition to support plant growth.

In our study, there was no consistent effect of eCO_2_ on net ECM production across the year (Fig. 4*A*, RR = 0.15); however, the difference in production across seasons between aCO_2_ and eCO_2_ was striking. Previous work in young forests regarded increased root length colonization by ECM fungi under eCO_2_ as an indication of greater C investment in nutrient acquisition (47), although increased ECM production is not consistent under eCO_2_ (46). The high ECM biomass production during the growing season herein indicates C investment into mycorrhizal associations during periods of rapid growth, and this was enhanced under eCO_2_ by 17% in the late growing season, partially disproving our hypothesis of consistent investment in do-it-yourself branching and outsourcing exudation over ECM. Conversely, there was a significant trend toward lower ECM biomass production under eCO_2_ compared to aCO_2_ in winter (*P* = 0.037). Greater ECM biomass production promotes nutrient acquisition from otherwise physically or chemically inaccessible nutrient sources (48) and generally peaks toward autumn coinciding with fruiting body production, but also with high tree nutrient demands (42). It is notable that high ECM biomass production coincides with leaf fall, as ECM fungi excrete enzymes capable of degrading complex organic N compounds (44) to release and capture nutrients from decomposing litter (49).

To compare C investment in different nutrient acquisition mechanisms, we calculated the relative magnitude of responses to eCO_2_ during the study period (Fig. 1) and contrasted C investment among mechanisms using correlation analysis. The comparison of response ratios (RR = 0 indicates no response; RR = 1 indicates an *e*-fold (2.7-fold) increase under eCO_2_, etc.) revealed the largest relative increase in investment under eCO_2_ in annual ECM turnover (RR = 1.29; 3.6-fold), followed by increased fine root branching in the organic horizon (RR = 0.55; 1.7-fold) and C exudation (RR = 0.26; 1.3-fold). Correlation analysis suggests that ECM biomass production declined with increasing root C exudation (*SI Appendix*, Fig. S6). This supports our hypothesis of a trade-off in investment between these two outsourcing nutrient acquisition strategies (15, 50); a trade-off which appears to become more severe (i.e., the correlation gradient steepens) under eCO_2_. It is conceivable that the more pronounced seasonal changes in exudation rates and ECM biomass under eCO_2_ reflect shifts in tree C investment to improve nutrient acquisition relative to seasonality in tree growth periods. Accordingly, high C investment in root exudates early in the growing season would prime SOM mineralization by heterotrophic microbes (15), whereas greater C investment in ECM biomass production later in the year would boost nutrient acquisition from decomposing litter and in soil microsites into which fine roots cannot extend. Exudation may be considered a do-it-yourself strategy, via the release of organic acids and enzymes to promote nutrient acquisition; however, the surface organic-rich nature of forest soils, alongside the observed upregulation of N transformation in root-free soil (12), supports our assignment of root exudation as predominantly an outsourcing strategy. The increase in C relative to N in exudation indicates exudation as an outsourcing strategy, releasing C to prime the microbial community. Under eCO_2_, net ECM biomass production declined with increasing root branching (*P* = 0.027; *SI Appendix*, Fig. S7), while root C exudation increased with branching (*P* = 0.047; *SI Appendix*, Fig. S7), which is commonly observed (51), suggesting consistent increased investment in do-it-yourself nutrient acquisition. These combined mechanisms demonstrate that tree nutrient acquisition strategies under eCO_2_ will be complex, with switching investment between do-it-yourself and outsourcing strategies according to nutrient demands and nutrient inputs into the forest. Combined increases in nutrient acquisition strategies belowground evidenced herein may promote SOM turnover belowground (13, 46), which feedback on soil respiration, as observed in this FACE experiment in the initial years after eCO_2_ (11). Notably, the observed increased in root exudation under eCO_2_ will promote SOM turnover and reduce soil C sequestration capacity (37). Provided nutrients are not lost from the system, the stoichiometric differences between plant biomass and SOM can still yield a C sink, especially when wood production is stimulated (9), although soil C losses via respiration will offset some C gains in aboveground biomass.

## Conclusions

Under eCO_2_, there are demonstratable seasonal increases in the investment in the complementary outsourcing nutrient acquisition strategies of root exudation and ECM biomass, alongside consistent investment in the do-it-yourself strategy of fine-root branching. The dynamic response of these nutrient acquisition strategies demonstrates that mature trees can exhibit plasticity in support of sustained additional growth in a mature temperate forest under eCO_2_. However, the relative investment of C in different strategies differs depending on the seasonal nutrient requirements, balancing C allocation to growth against nutrient acquisition. The seasonal changes in exuded compounds maintain support for nutrient acquisition while also reflecting tree N conservation. Our findings provide a representation of the mechanisms by which a mature temperate forest can acquire nutrients across growing phases and how this will change as atmospheric CO_2_ increases. Changes in the allocation of C belowground, driven by the CO_2_ fertilization effect, will have implications for long-term C sequestration, SOM turnover, and overall C storage in forests under rising atmospheric CO_2_.

## Methods

### Site Description.

The University of Birmingham’s BIFoR FACE experiment was established in 2016 in a mature, temperate forest located in central England, United Kingdom (52° 48’ 3.6” N, 2° 18’ 0”W) (29). The canopy is dominated by *Quercus robur* (pedunculate oak; Linnaeus), which are over 175 y old and comprise 92% of total basal area (9). The soil is classed as a dystric Cambisol with a sandy-clay texture and a pH of 4.5 in the O horizon (52). The experimental design includes six FACE arrays (diameter *c*. 30 m); three are eCO_2_ (actual eCO_2_ treatment achieved between 2017 to 2022 = 140 ± 38 ppm above ambient) and three aCO_2_ arrays (29). Starting 3 April 2017, eCO_2_ has been supplied from oak bud burst (~1 April) to leaf fall (~1 November) between dawn and sunset (29).

### Root Exudate Collection and Analysis.

Root exudates were collected in situ from intact *Q. robur* root systems in the O horizon four times between 2020 and 2021 (10 to 11th August 2020, 11 to 12th November 2020, 3rd to 4th March 2021, and 10 to 11th June 2021), using a method adapted from ref. 53. Three oak trees per array were randomly selected and root boxes with Perspex windows were installed within 1 m of each tree (*SI Appendix*, Fig. S1) in August 2020. Due to the COVID pandemic, it was not possible to install the root boxes in sufficient time to permit regrowth for the first sampling point. Therefore, roots from the same tree were excavated from the O-horizon and used for exudate collection. The root boxes with two windows were then installed for all subsequent root exudate collections, which alternated between windows to allow root recovery from disturbance. Oak roots were identified based on surveys of root morphology for oak and understory trees outside the experimental arrays, where it was possible to trace roots back to individuals. We focused on oak roots as oak represents 92% of the total basal area (9).

Two root systems per box were used for exudate collection, giving pseudoreplication of *n* = 6 per array. The fine root systems (approx. 10 cm length, diameter <2 mm) were carefully excavated. Once excavated, the root systems were washed with a carbon-free nutrient solution (NH_4_NO_3_ 40 mg L^−1^; KH_2_PO_4_ 13.6 mg L^−1^; K_2_SO_4_ 349 mg L^−1^; CaCl_2_ 441 mg L^−1^; and MgSO_4_.7H_2_O 0.3705 g L^−1^) and tweezers were used to gently break up soil aggregates that could then be removed from the roots by washing. After washing, the intact roots, still attached to the tree, were placed in a glass syringe filled with glass beads (750 μm diameter, 30 g) and nutrient solution (10 mL) to mimic soil, with the syringe plugged with a three-way valve at the base to permit exudate collection (*SI Appendix*, Fig. S1). In addition, one blank syringe (i.e., without roots) was incubated in each root box to correct for any background C and N input. Roots were allowed to recover and acclimatize for 48 h, with the nutrient solution replaced after 24 h. After the 48-h recovery period, the syringe was washed with a C- and N-free nutrient solution (3 × 10 mL of KH_2_PO_4_ 13.6 mg L^−1^; K_2_SO_4_ 349 mg L^−1^; CaCl_2_ 441 mg L^−1^ and MgSO_4_.7H_2_O 0.3705 g L^−1^), a final 10 mL of nutrient solution was added and exudates were captured during 24 h. This time period accounted for the diurnal release of exudates but also included potential reuptake of compounds; the amount of exudates collected therefore represents net exudation, reported as mass of exudate per unit mass of root. After 24 h, the solution was collected, and the roots were washed with a further 2 × 10 mL of C- and N-free nutrient solution. Roots were immediately cut level with the glass beads. The exudate solution was analyzed for dissolved oxidizable carbon (DOC; assumed herein to be organic) and total dissolved nitrogen (TDN; organic and mineral N) by combustion oxidation (TOC-L Organic Carbon Analyzer, Shimadzu, Japan); the relative SD was <1% for DOC and <4% for TDN. Specific root exudation based on the mass and area of the root used for exudate collection. Areal root exudation was calculated using the closest estimate of root standing stock for each collection point, using the sum of <1 mm and 1 to 2 mm, as root scanning indicated both were present in the exudate collection.

Roots used for exudate collection were immediately scanned to quantify root length, diameter, surface area, and branching, using RhizoVision Explorer version 2.0.2 (54) to calculate branching frequency (per mm) and SRL = root length/root dry mass. Root diameters ranged from 0.15 to 0.35 mm for 1st order, 0.31 to 0.46 mm for 2nd order, 0.56 to 0.96 mm for 3rd order, and 1.05 to 1.5 mm for 4th order roots. Roots were frozen and lyophilized to determine dry mass and total C and N content via combustion on an organic elemental analyzer (vario PYRO cube, Elementar Analysensysteme GmbH, Hanau, Germany). The instrument was calibrated with sulfanilamide (N: 16.26%, C:41.81%, S: 18.62%) and the precision as a relative SD was <5% for both C and N.

### LC–MS Analysis of Roots and Root Exudates.

Roots and exudates collected in August 2020 were further analyzed for their composition by LC-MS. Roots and exudates (10 mL) were lyophilized, and dissolved in methanol:water (30:70 *v*/*v*) containing 0.01% formic acid. Roots metabolites were extracted by using metal balls in a mixer for 3 min at 30 Hz and then centrifuged (13,000 rpm, 20 min at 4 °C). Exudates were diluted in the same solvents (methanol:water 30:70 *v*/*v* containing 0.01% formic acid). The supernatant was filtered with 0.22 µm filters of regenerated cellulose (Phenomenex). An aliquot of 5 µL was injected into in the ACQUITY UPLC I-Class System and a SYNAPT G2-S high-definition mass Spectrometer MS/MS detector (Waters®). Separation of compounds by LC was set by a Kinetex 2.6 µm EVO C18 analytical column (50 × 2.1 mm, Phenomenex) using a gradient of methanol and H_2_O supplemented with 0.01% formic acid. Extracts were analyzed in both modes of ionization, positive and negative. After data acquisition, raw data were transformed into .CDF with Databridge software and processed with in-house R scripts using R studio (55).

### Mycorrhizae Ingrowth and Turnover Quantification.

Mycorrhizal extramatrical hyphal production was determined using nylon mesh (45 μm) bags to exclude roots but permit hyphal ingrowth. The bags (3 cm × 10 cm) were filled with coarse, acid-washed sand and heat sealed. Three bags were installed at a 45° angle in the O horizon at five locations per array. The first set of bags was installed on 19th May 2020 and each of the three bags was collected and replaced at increasing time intervals: One bag was collected and replaced every 3 mo (19th August 2020, 16th November 2020, 23rd February 2021, and 24th May 2021), the second bag was collected and replaced every 6 mo (16th November 2020 and 24th May 2021) and the final bag was left in place for the full 12-mo duration (24th May 2021). This staggered collection provided estimates of production and turnover of hyphae (45). Once the bags were removed, the contents were immediately split into subsamples; one was frozen at −20 °C for ergosterol extraction and one subsample (1 g) as used to determine moisture content (105 °C, 24 h).

To determine ECM biomass, ergosterol was extracted using a protocol adapted from ref. 42, assuming ECM was the main source of ergosterol in ingrowth bags. Sand from each bag (5 g) was extracted with 10% KOH (4 mL) and cyclohexane (1 mL), sonicated for 15 min, and then extracted using an orbital shaker for 45 min (200 strokes min^−1^). The extract was then heated in a water bath at 70 °C for 90 min. Phase separation was exerted by addition of e-pure water (1 mL) and cyclohexane (2 mL), followed by centrifugation (5 min, 4,000 rpm). The cyclohexane was transferred and the phase separation repeated. The dried extract (gentle flow of N_2_ at 40 °C) was redissolved in methanol (400 μL) and filtered (0.45 μm Teflon syringe filters). The extract was subsequently analyzed for ergosterol using high-performance liquid chromatography system (UltiMate 3000, Thermo Fisher Scientific, United Kingdom), fitted with a C_18_ reverse phase column (250 mm × 4.6 mm, 5 μm i.d.), preceded by a C_18_ guard column. The solvent program used a 100% methanol mobile phase at a rate of 1.0 mL min^−1^. Ergosterol detection was performed at 282 nm using a UV detector. Quantification was achieved by external calibration using an ergosterol standard, and the ergosterol content converted to fungal biomass based on 3.3 µg ergosterol mg^−1^ fungal biomass (56).

### Calculation of Hyphal Turnover and RR to eCO_2_.

Seasonal ECM hyphal production rates were determined using fungal biomass based on ergosterol content in ingrowth bags that were collected every 3 mo, normalized to installation duration. In addition, the staggered collections at 6 and 12 mo were used to calculate turnover of ECM hyphal biomass:

$$\mu =\frac{-\ln\left(\frac{B_{12}-B_{6b}}{B_{6a}}\right)}{t_{2}-t_{1}},$$

where μ is the turnover coefficient, B_12_ is the biomass present in the ingrowth bag after 12 mo (t_2_), B_6b_ is the biomass of the 6-mo ingrowth bag collected at t_2_, and B_6a_ is the biomass in the ingrowth bag collected at 6 mo at t_1_ (45).

To compare the strength of eCO_2_ treatment effects across different response variables, we calculated the annual RR for root traits, root exudation, and hyphal production and turnover as

$$\text{RR}=\text{ln}\left(\frac{R_{e}}{R_{a}}\right),$$

where R_e_ is the mean annual response under eCO_2_, and R_a_ is the mean annual response under aCO_2_. The variance (v) of RR was calculated as

$$v=\frac{{\sigma_{e}}^{2}}{n_{e}{R_{e}}^{2}}+\frac{{\sigma_{a}}^{2}}{n_{a}{R_{a}}^{2}},$$

where σ_e_ and σ_a_ are the SD, and n_e_ and n_a_ are the sample sizes for the eCO_2_ and aCO_2_ treatments, respectively (57).

### Statistical Analyses.

All statistical analyses and visualization were conducted using R v4.4.0 (55). Data were checked for normality using QQ plots, and for heteroscedasticity by plotting residuals vs. fitted values, and the data were log transformed if necessary. To evaluate the effect of eCO_2_ on exudate, root morphology and nutrient content, and ECM biomass production, we used linear mixed effect models [*mixed* function in the package *afex* (58)], comparing a full model with CO_2_ treatment as a fixed effect, and date and array as random effects, with a null model including with date and array and random effects only by one-way ANOVA. To determine seasonal effects, the best fit model was then compared to a model with array only as a random effect. Where the model including CO_2_ treatment was significant, a *P* value for the treatment effect was determined using the Kenward–Roger method. In addition, for ECM biomass production, Wilcoxon rank-sum tests were used to determine eCO_2_ treatment effects at individual timepoints. We then assessed possible trade-offs among C investment in different mechanisms using Pearson correlation networks for roots and exudates with a threshold defined at 0.5. Correlations between root C exudation or root traits and mycorrhizal fungal production were determined using mean values for each array (*n* = 5 for mycorrhizal fungi and *n* = 6 for exudates).

For the August 2020 collection, roots and exudates were further analyzed via LC–MS for untargeted metabolomics. Feature abundances were normalized to the dry mass of root used for exudate collection. Differences were visualized by PCA using the *vegan* package (59). Data were log-transformed and scaled to unit variance prior to separate PCAs for features detected in exudates and roots under positive and negative ionization. The first two principal components were then plotted to visualize differences between the metabolite profiles under aCO_2_ and eCO_2_ using the *factoextra* package (60). Differences in individual features between aCO_2_ and eCO_2_ were determined using the Kruskal–Wallis test, as assessments of normality using skewness and kurtosis indicated that the data were not normal and resistant to transformation.

## Supplementary Material

Appendix 01 (PDF)

## Data Availability

Root exudate, root morphology and mycorrhizal turnover datasets have been deposited in NERC Environmental Information Data Centre (10.5285/a05b9519-f0c8-48ef-a9c6-43d0326f590f) (61).
