# Supplementary material for: Elevated CO2 alters relative belowground carbon investment for nutrient acquisition in a mature temperate forest
Source: Proc Natl Acad Sci U S A. 2025 Jul 15;122(29):e2503595122. doi: 10.1073/pnas.2503595122 (PMC12304975; doi:10.1073/pnas.2503595122)

Elevated CO_2 alters relative belowground carbon investment for nutrient acquisition in a mature temperate forest

Michaela K. Reay[a,b,c*], Emma J. Sayer[d,e], Andrew Smith[f], Victoria Pastor[g], Angeliki Kourmouli[b,d], Miles Marshall[f], Robert T. Grzesik[a,b], Iwan Evans[a,b], Manon Rumeau[a,b], Kris Hart[b], Jiaojiao Ma[a], Richard J. Norby[a,b,h], A. Robert MacKenzie[a,b], R. Liz Hamilton[a, b], Iain P. Hartley[i], Sami Ullah[a,b]

[a] School of Geography, Earth and Environmental Science, University of Birmingham, B15 2TT, UK

[b] Birmingham Institute of Forest Research, University of Birmingham, Birmingham, B15 2TT, UK

[c] Organic Geochemistry Unit, School of Chemistry, University of Bristol, BS8 1TS, UK

[d] Lancaster Environment Centre, Lancaster University, Lancaster, LA1 4YQ, UK

[e] Institute of Botany, Ulm University, D-89081, Ulm, Germany

[f] School of Environmental and Natural Sciences, Bangor University, Bangor, LL57 2UR, UK

[g] Department of Biology, Biochemistry, and Natural Sciences, School of Technology and Experimental Sciences, Universitat Jaume I, 12006 Castelló de la Plana, Spain

[h] Environmental Sciences Division, Oak Ridge National Laboratory; Oak Ridge, TN, 3 7830, USA

[i] Geography, Faculty of Science, Environment and Economy, University of Exeter, Exeter, EX4 4RJ, UK

Corresponding author (*): Michaela K. Reay, michaela.reay@bristol.ac.uk

Additional author for correspondence: S. Ullah s.ullah@bham.ac.uk

Figure S1: Root boxes and root exudate collection photo A root box design with two Perspex windows, a hollow base that was covered in chicken wire to prevent animal burrowing, and a removal lid to allow access. B in situ root box, with insulation on windows installed to a depth of 30 cm at BIFoR FACE within 1 m of an oak tree. C Collection of root exudates using root boxes.

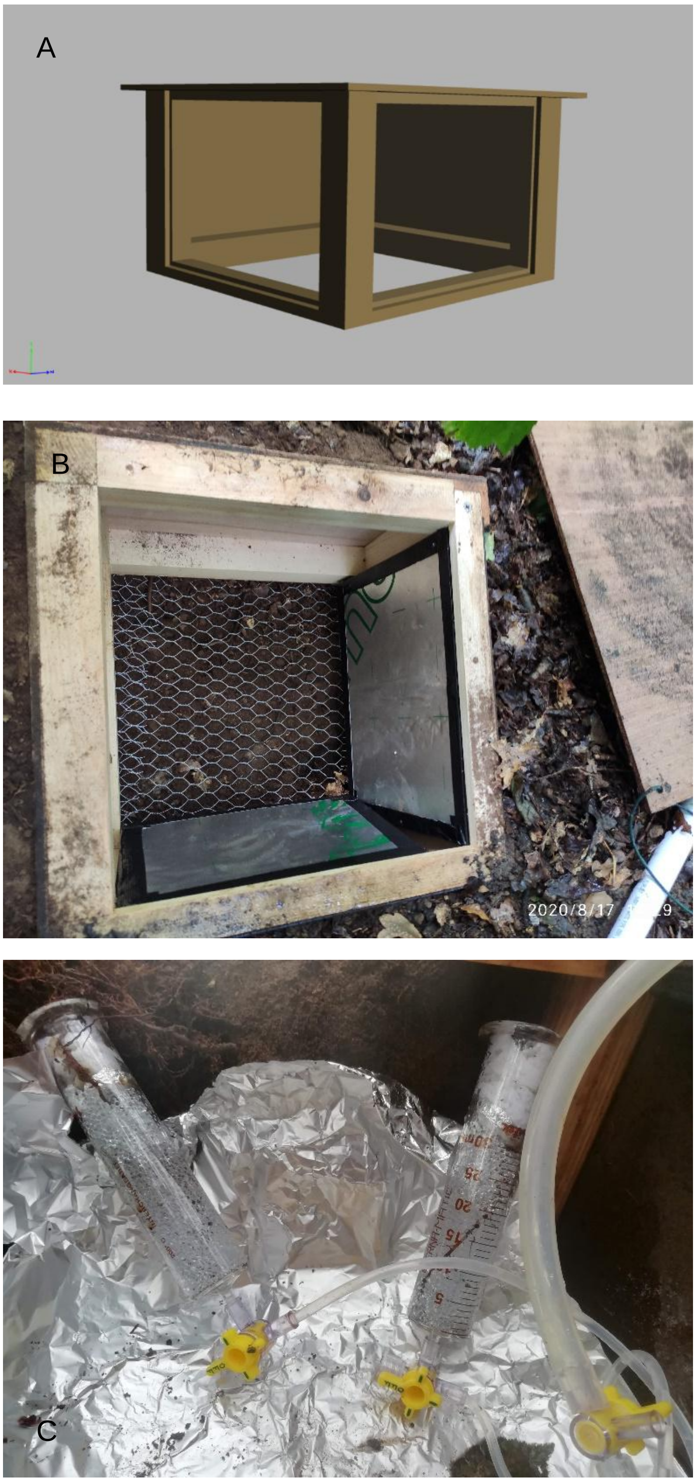

Figure S2: Root exudation by area of mature oak trees under ambient (aCO_2) and elevated CO_2 (eCO_2), showing (A) C exudation and (B) N exudation. Boxes denote the 25th and 75th percentiles and median lines are given for *n*=18 pseudo-replicates, whiskers indicate values up to 1.5x the interquartile range.

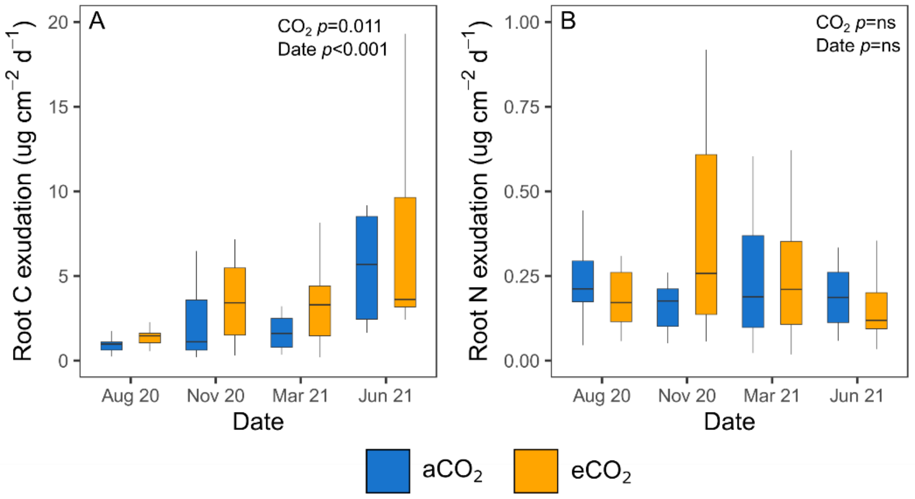

Figure S3: Pearson correlation matrix of root exudation and root morphology variables for roots and exudates collected at four time points between 2020 to 2021 under ambient and elevated CO_2. Significant (p<0.05) positive and negative correlations are indicated by blue and red circles, respectively

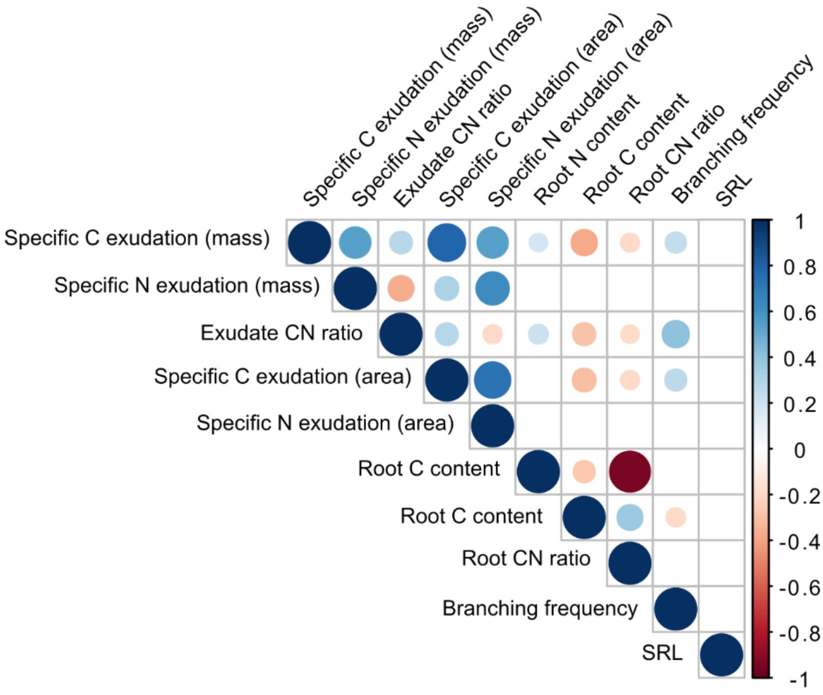

Figure S4: Root standing stock in the O horizon at BIFoR-FACE under ambient (aCO_2) and elevated CO_2 (eCO_2) between 2020 and 2021, showing (A) roots <1 mm and (B) roots 1-2 mm. Values are mean ± SE (*n*=3). Five pseudo-replicates were collected per array. The closest estimate of root standing stock was used to calculate total exudation rate for each collection point, using the sum of <1 mm and 1-2 mm, as root scanning indicated both were present in the exudate collection.

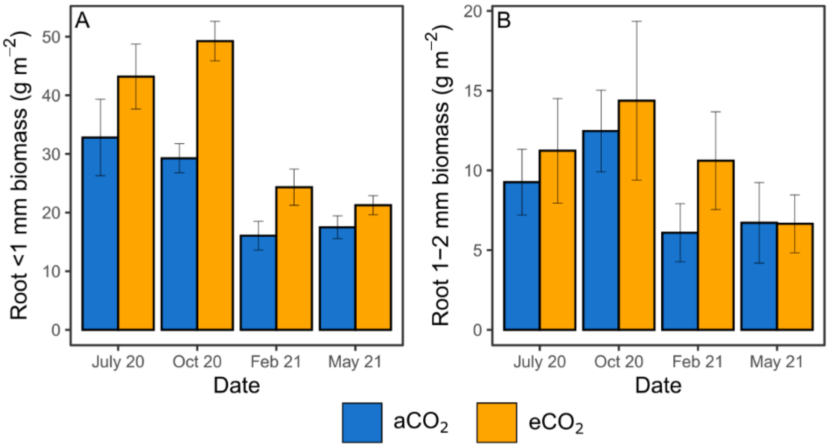

Figure S5: Root nutrient content of mature oak trees under ambient (aCO_2) and elevated CO_2 (eCO_2) for roots used for exudate collection between 2020 and 2021 showing (A) total carbon, B nitrogen and C root C:N ratio for roots used for exudate collection. Boxes denote the 25th and 75th percentiles and median lines are given for *n*=18 pseudo-replicates for each treatment (true replication *n*=3), whiskers indicate values up to 1.5x the interquartile range.

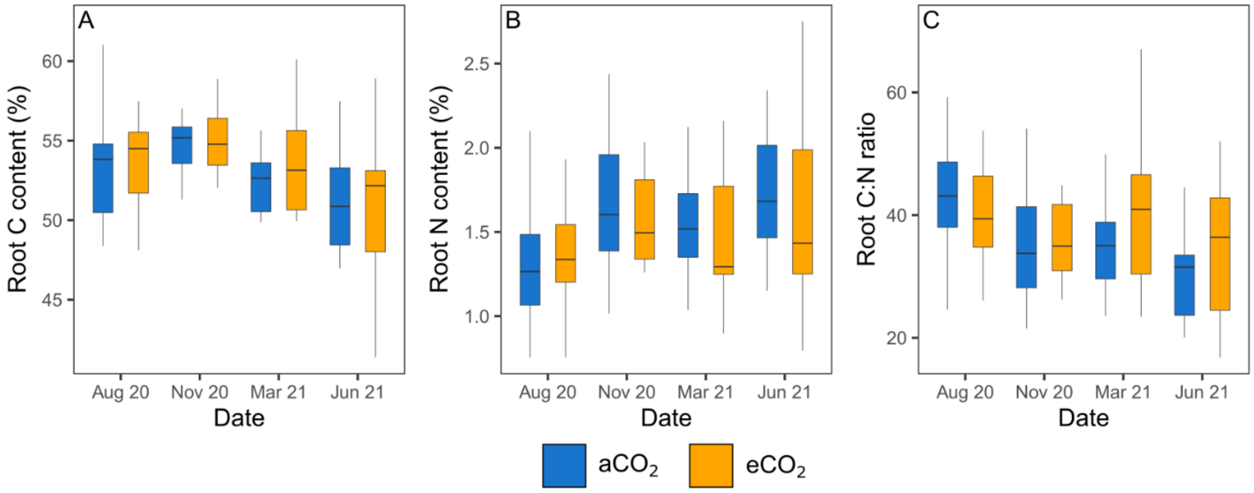

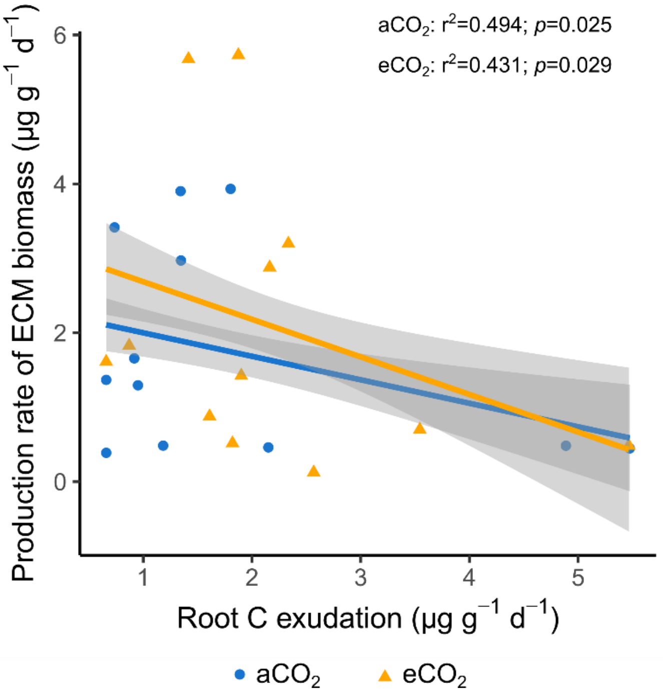

Figure S6: Correlation of mycorrhizal fungi production rates and root C exudation, show (A) ECM and (B) AMF production rates for four timepoints in 2020-2021. Each filled symbol is the ECM production rate compared to the C exudation rate at array level (pseudo replication n=5 and 6, respectively for each data point). Lines indicate linear regressions for the aCO_2 and eCO_2 treatments, and shaded areas indicate 95% confidence intervals.

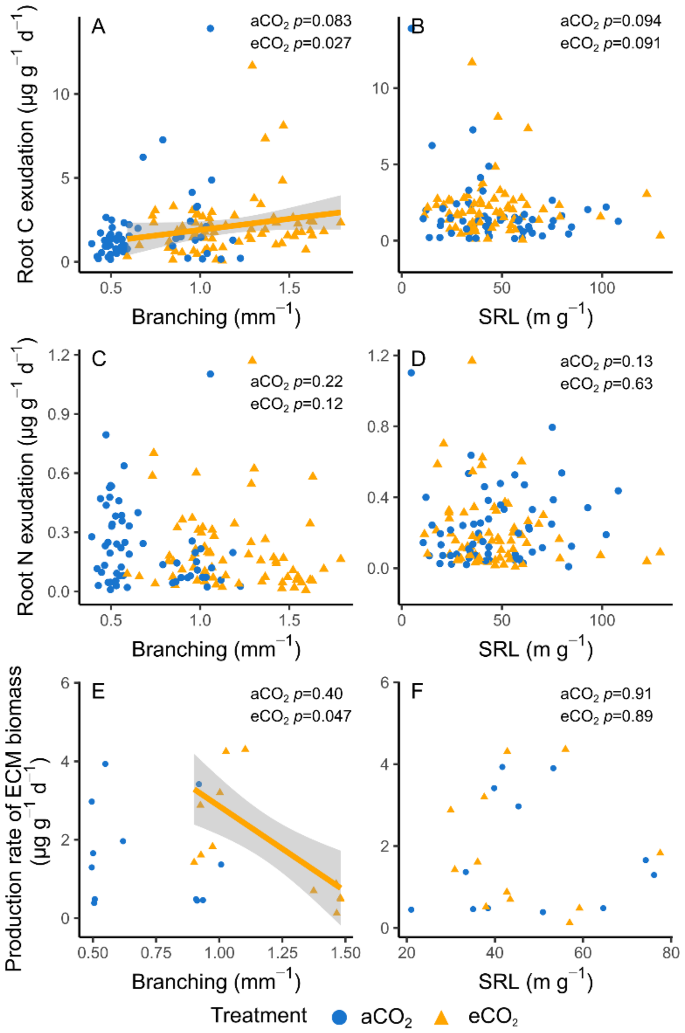

Figure S7: Correlation of root traits (branching frequency and specific root length (SRL)) and root C exudation (A, B), root N exudation (C, D) and ECM biomass production (E, F) across four timepoints in 2020-2021. Each filled symbol in parts A-D is C or N exudation rate compared to branching or SRL for each pseudo replicate (*n*=18) at each timepoint. For parts E and F, each filled symbol is the ECM production rate compared to root branching or SRL array level (*n*=5 and 6, respectively for each data point). Lines indicate linear regressions for the aCO_2 and eCO_2 treatments, and shaded areas indicate 95% confidence intervals. Only significant (*p*<0.05) correlations are shown.

Table S1: Results of comparisons between mixed effects models for root, exudate and mycorrhizal fungal parameters analysed between 2020 to 2021 at four timepoints to determine the effect of elevated CO_2 and seasonal variation. Prior to mixed effects models, data were checked for normality, via QQ plots, and heteroscedasticity, by plotting residuals vs. fitted values, and the data were log transformed, if it was not normal as indicated in the Parameter column. Model A tested treatment (eCO_2, aCO_2) as a fixed effect with date and array as random effects, the Null model tested only collection date and array as random effects. Array was included as a random effect to account for potential spatial variability across the forest. Firstly, model A vs. Null were compared (One-way ANOVA) to determine if treatment had a significant effect. If $p<0.05$, Model A was selected, indicating treatment had a significant effect. If $p>0.05$, the Null model was selected, indicating the treatment was not significant. Subsequently, the best model from either model A or the Null model was then compared to the equivalent model with only array as a random effect. This tested whether there was a significant date effect, either when there was a treatment effect (A vs. B) or if there was not a treatment effect (Null vs. Null B), using a One-Way ANOVA. For both A vs. B and Null vs. Null B, if $p<0.05$, this indicated date had a significant effect, while $p>0.05$ indicated that date did not have a significant effect. For each parameter, the best fit model is highlighted in bold.

Parameter	Model	Fixed effects	Random effects	Treatment *p*	Comparison model	AIC	X^2	One way ANOVA *p*
Log(Branching)	A	Treatment	Date, array	<0.001	Null	-25.959	12.884	<0.001
					B	-25.959	101.25	<0.001
	Null	1	Date, Array	-	A	-15.075	-	-
	B	1	Array	-	A	71.288	-	-
Log(SRL)	A	Treatment	Date, array	0.934	Null	212.93	0	-
	Null	1	Date, array	-	A	208.02	-	1
					Null_B	208.02	-	-
	Null_B	1	Array	-	Null	71.288	0	1
Root TC	A	Treatment	Date, array	0.45	Null	657.53	1.3195	0.025
	Null	1	Date, array	-	A	656.85	-	-
					Null_B	656.85	14.611	<0.001
	Null_B	1	Array	-	Null	669.46	-	
Root TN	A	Treatment	Date, array	0.0384	Null	129.84	0	1
	Null	1	Date, array	-	A	125.26	-	
					Null_B	125.26	6.011	0.014
	Null_B	1	Array	-	Null	129.27	-	
Root CN	A	Treatment	Date, array	0.476	Null	955.62	2.8597	0.059

	Null	1	Date, array	-	A	953.76	-	-
					Null_B	953.76	7.078	0.008
	Null_B	1	Array	-	Null	955.62	-	
Log(TOC mass)	A	Treatment	Date, array	0.037	Null	317.81	3.5499	0.011
					B		643.16	<0.001
	Null	1	Date, array	-	A	318.36	-	-
	B	Treatment	Array	0.026	A	958.96	-	-
Log(TDN mass)	A	Treatment	Date, array	0.665	Null	351.46	0.6115	0.945
	Null	1	Date, array	-	A	347.83	-	
					Null_B		25.58	<0.001
	Null_B	1	Array		Null	371.41	-	-
Log(CN)	A	Treatment	Date, array	0.021	Null	260.35	3.3713	0.024
					B		74.739	<0.001
	Null	1	Date, array		A	262.35		
	B	Treatment	Array	0.041	A	335.72		
Log(TOC area)	A	Treatment	Date, array	0.011	Null	330.62	4.5344	0.021
					B		37.746	<0.001
	Null	1	Date, array	-	A	338.15	-	-
	B	Treatment	Array	0.012	A	374.36	-	
Log(TDN area)	A	Treatment	Date, array	0.981	Null	345.07	0.312	0.984
	Null	1	Date, array	-	A	341.55	-	-
					Null_B		0.312	0.845
	Null_B	1	Array	-	Null	339.55	-	-
Log(ECM production)	A	Treatment	Date, Array	0.548	Null	533.40	0.8952	0.344
	Null	1	Date, Array	-	A	532.51	-	-
					Null_B	532.51	29.343	<0.001
	Null_B	1	Array	-	Null	559.74	-	-

Table S2: Summary of features identified in untargeted metabolomic analysis of exudates and roots under ambient (aCO_2) and elevated CO_2 (eCO_2) in August 2020. Upregulated and downregulated features under eCO_2 relative to aCO2 were determined following normalization to the root mass used for exudate collection. The Kruskal-Wallis test was used to determine if features were significantly different (p<0.05) between the aCO_2 and eCO_2 treatments, as assessments of normality using skewness and kurtosis indicated the data were not normal.

Pool	Ionisation mode	Total number of ions	Total upregulated under eCO_2 (p<0.05)	Total downregulated under eCO_2 (p<0.05)
Exudate	Positive	4025	21	203
Exudate	Negative	2585	56	115
Root	Positive	3016	120	434
Root	Negative	5481	285	120

Table S3: Summary of common ions identified between the roots and exudates under ambient (aCO_2) and elevated CO_2 (eCO_2) in August 2020. Upregulated and downregulated ions in the roots and exudates under eCO_2 relative to aCO_2 were determined following normalization to the root mass used for exudate collection. The Kruskal-Wallis test was used to determine if ions were significantly different (p<0.05) with exudates or roots between the aCO_2 and eCO_2 treatments, as assessments of normality using skewness and kurtosis indicated the data were not normal.

Ionisation mode	Number of common ions	Upregulated in roots (p<0.05)	Downregulated in roots (p<0.05)	Upregulated in exudates (p<0.05)	Downregulated in exudates (p<0.05)
Positive	1565	3	7	0	10
Negative	1188	0	4	0	4

Extended data 2: Upregulated and downregulated features detected in exudates and roots in the August 2020 exudate collection.

Data background:

Each panel represents a feature which was accumulated or depleted under eCO_2 relative to aCO_2. This was assessed based on the abundance of a feature, normalised to the mass of root used for exudate collection. The Kruskal-Wallis test was used to determine if features were significantly different ($p<0.05$) with exudates or roots between the aCO_2 and eCO_2 treatments, as assessments of normality using skewness and kurtosis indicated the data were not normal. For presentation of data, values are normalised for each individual feature, as all values are relative abundance and not absolute concentrations. All analyses were untargeted with no compound identification, hence each individual feature which varied is distinguished by their unique *m*/*z* value (to 2 d.p.).

Figure S8: Unique features (to 2 d.p.) present in root exudates under positive ionisation that are significantly (Kruskal-Wallis, p<0.05) accumulated under eCO_2. Boxes denote the 25th and 75th percentiles and median lines are given for n=18 pseudo-replicates for each treatment (true replication n=3), whiskers indicate values up to 1.5x the interquartile range, and filled circles indicate outliers.

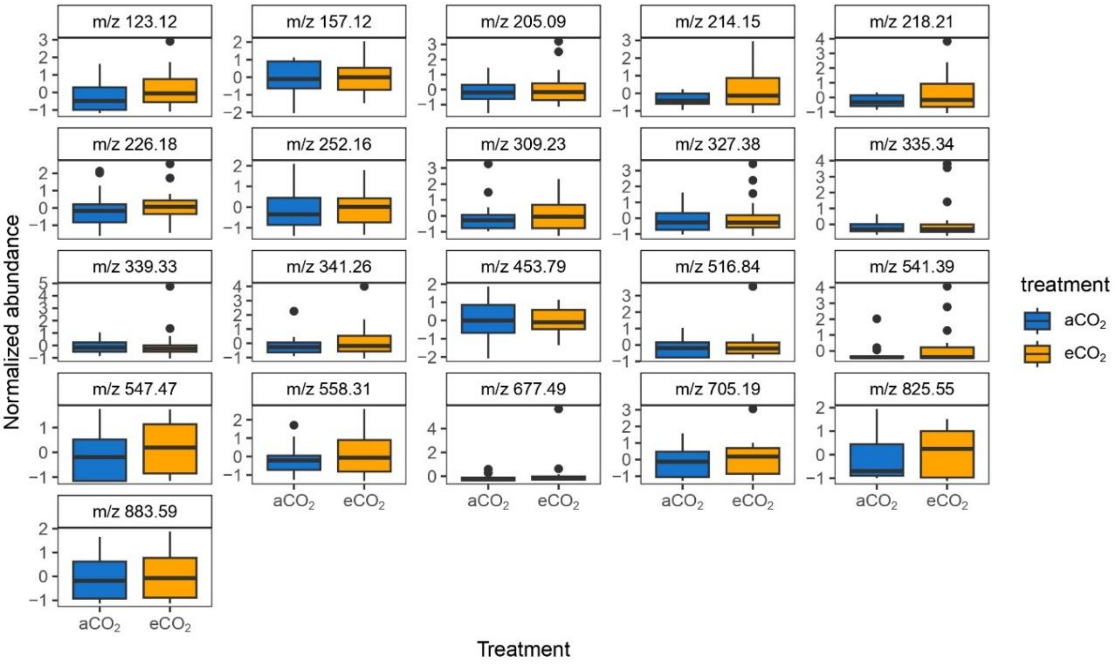

Figure S9a: Unique features (to 2 d.p.) present in root exudates under positive ionisation that are significantly (Kruskal-Wallis, $p<0.05$) depleted under eCO_2. Boxes denote the 25th and 75th percentiles and median lines are given for $n=18$ pseudo-replicates for each treatment (true replication $n=3$), whiskers indicate values up to 1.5x the interquartile range, and filled circles indicate outliers.

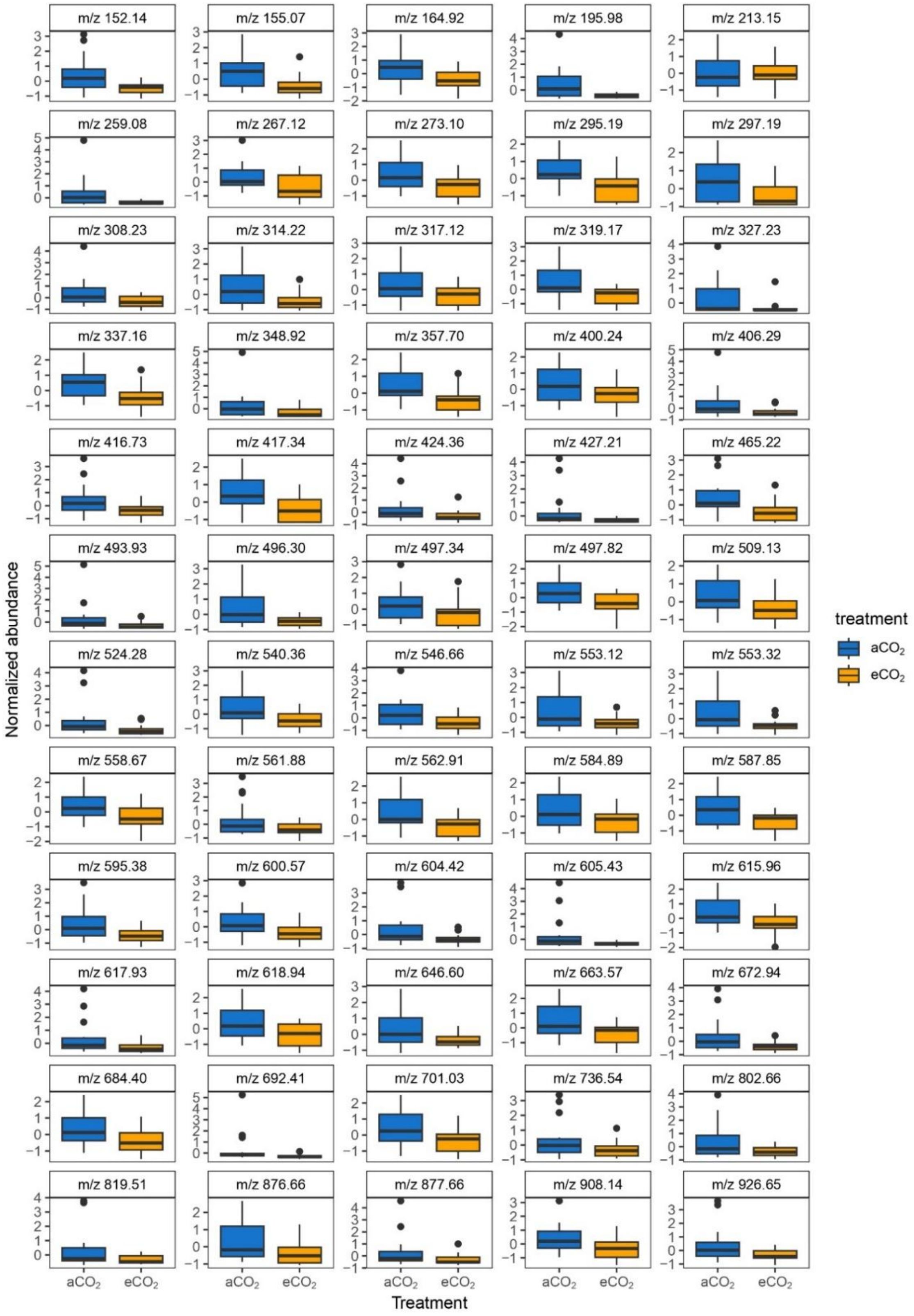

Figure S9b: Unique features (to 2 d.p.) present in root exudates under positive ionisation that are significantly (Kruskal-Wallis, p<0.05) depleted under eCO_2. Boxes denote the 25th and 75th percentiles and median lines are given for n=18 pseudo-replicates for each treatment (true replication n=3), whiskers indicate values up to 1.5x the interquartile range, and filled circles indicate outliers.

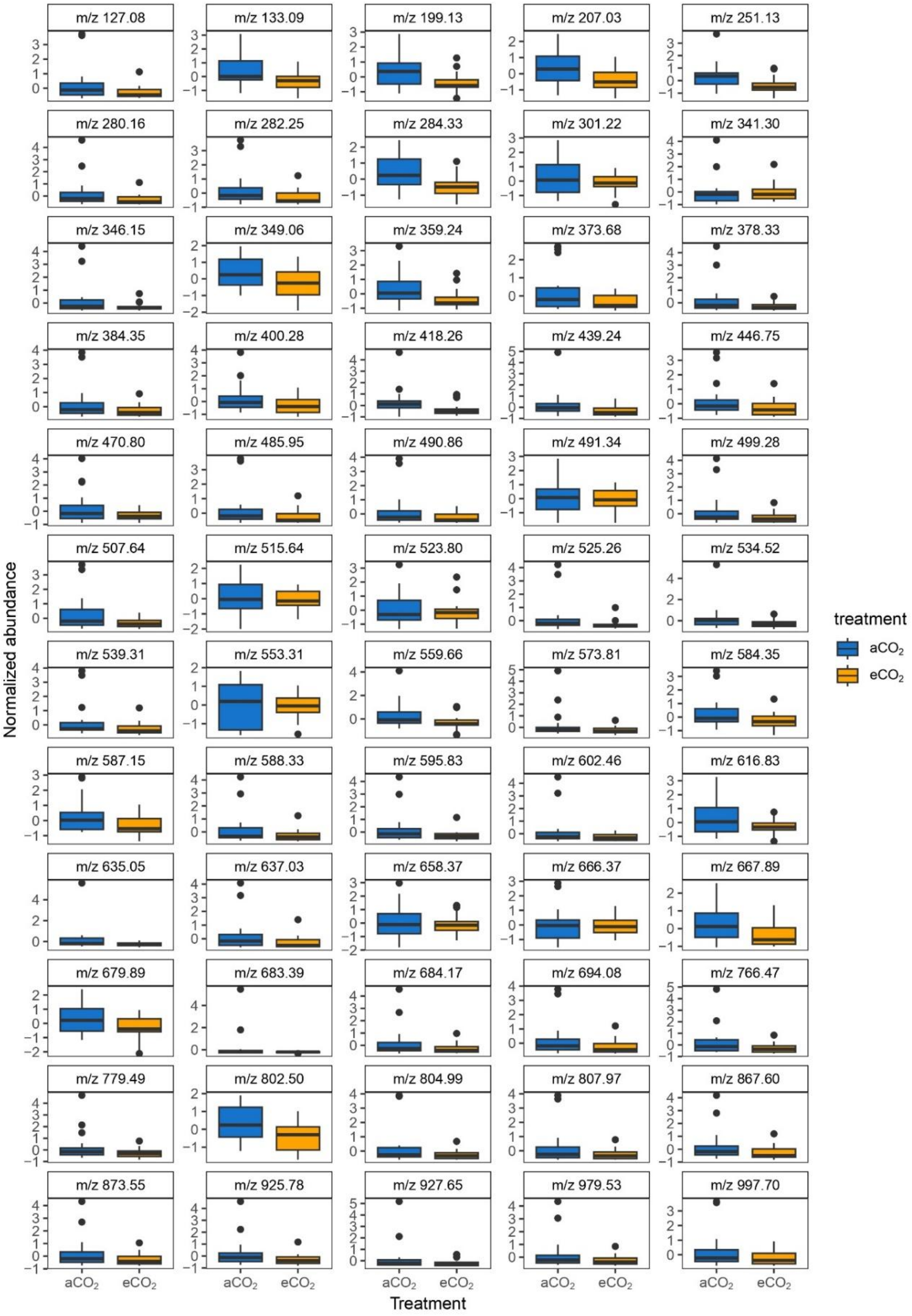

Figure S10: Unique features (to 2 d.p.) present in root exudates under negative ionisation that are significantly (Kruskal-Wallis, $p<0.05$) accumulated under eCO_2. Boxes denote the 25th and 75th percentiles and median lines are given for $n=18$ pseudo-replicates for each treatment (true replication $n=3$), whiskers indicate values up to 1.5x the interquartile range, and filled circles indicate outliers.

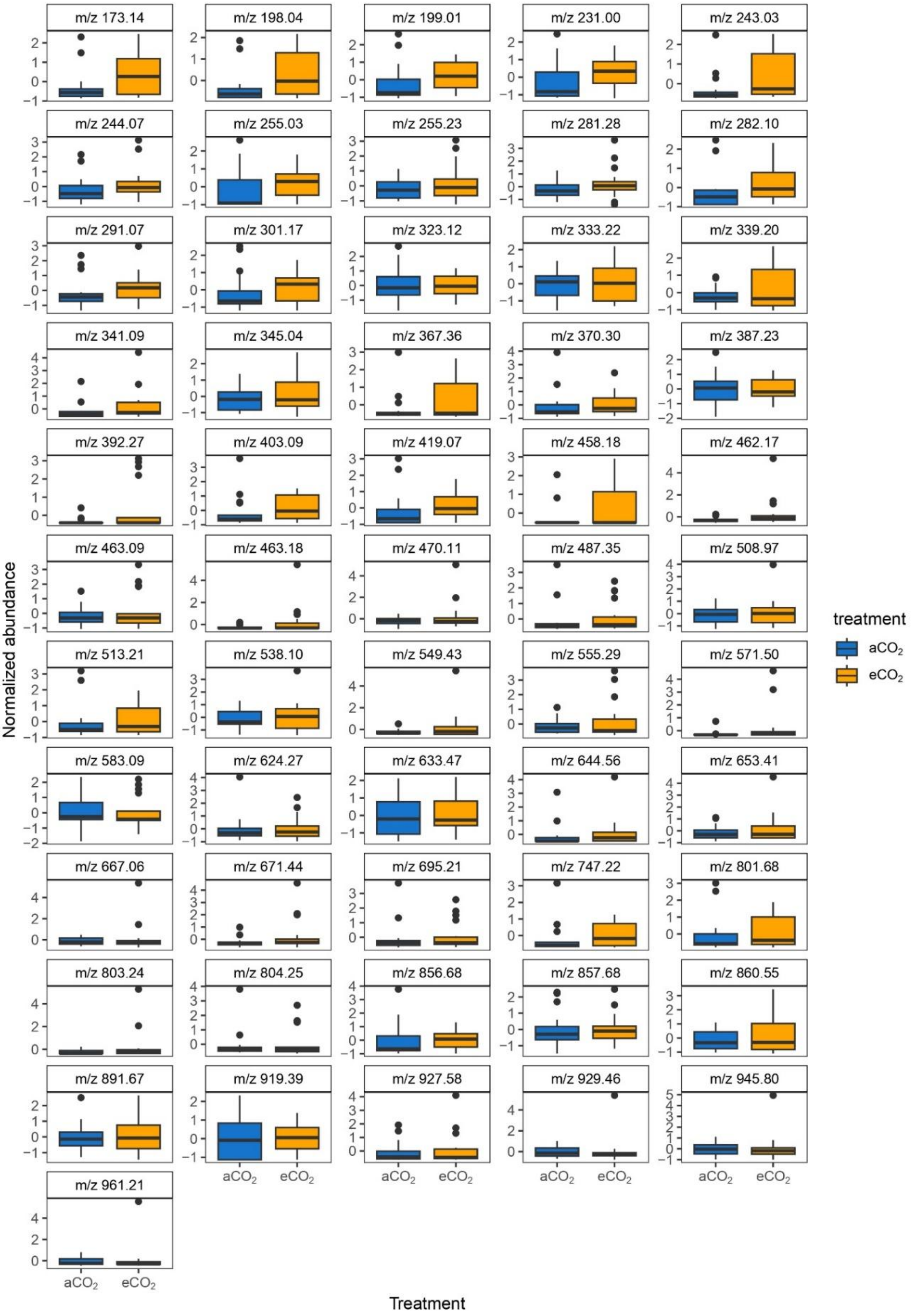

Figure S11a: Unique features (to 2 d.p.) present in root exudates under negative ionisation that are significantly (Kruskal-Wallis, $p<0.05$) depleted under eCO_2. Boxes denote the 25th and 75th percentiles and median lines are given for $n=18$ pseudo-replicates for each treatment (true replication $n=3$), whiskers indicate values up to 1.5x the interquartile range, and filled circles indicate outliers.

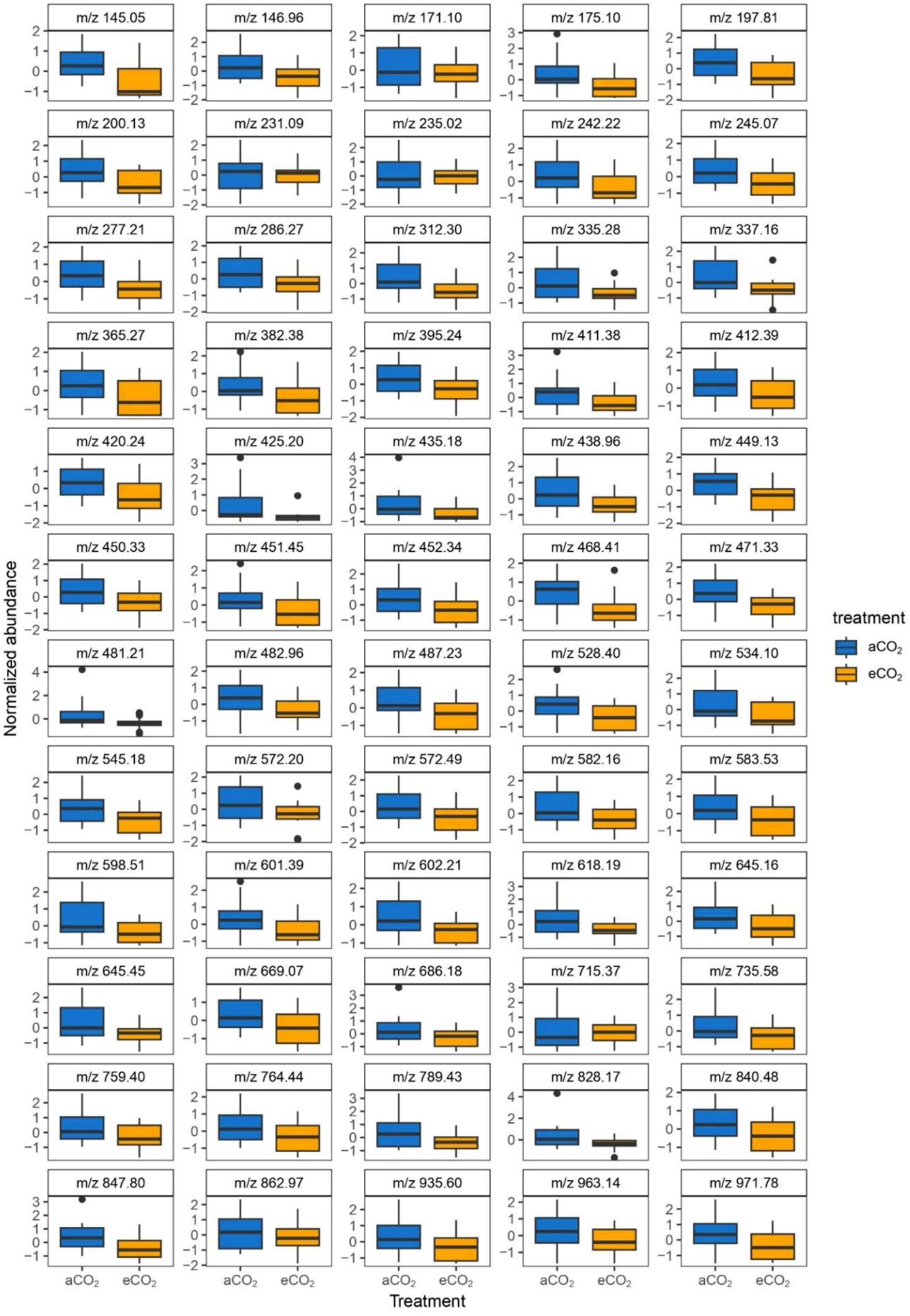

Figure S11b: Unique features (to 2 d.p.) present in root exudates under negative ionisation that are significantly (Kruskal-Wallis, p<0.05) depleted under eCO_2. Boxes denote the 25th and 75th percentiles and median lines are given for n=18 pseudo-replicates for each treatment (true replication n=3), whiskers indicate values up to 1.5x the interquartile range, and filled circles indicate outliers.

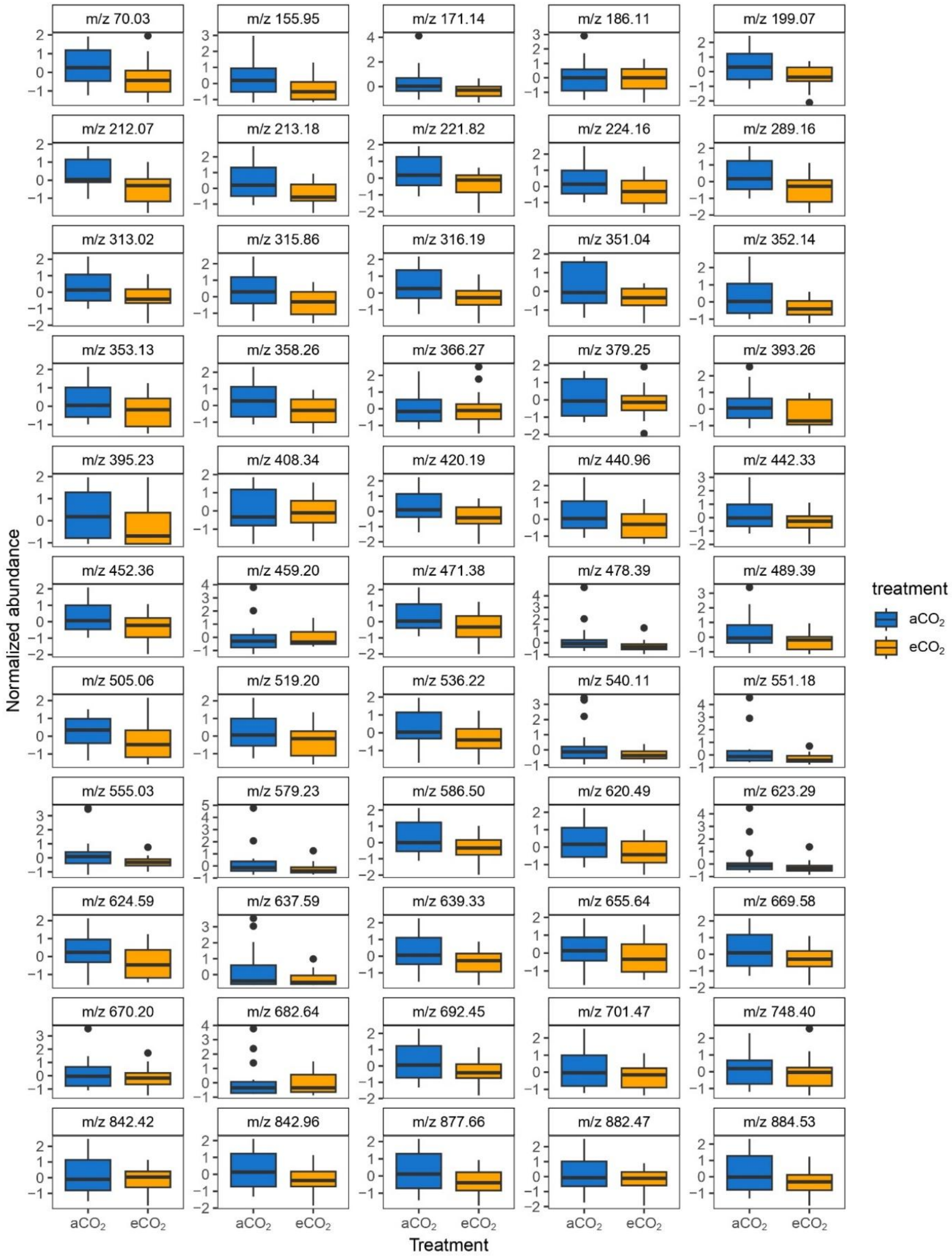

Figure S11c: Unique features (to 2 d.p.) present in root exudates under negative ionisation that are significantly (Kruskal-Wallis, $p<0.05$) depleted under eCO_2. Boxes denote the 25th and 75th percentiles and median lines are given for $n=18$ pseudo-replicates for each treatment (true replication $n=3$), whiskers indicate values up to 1.5x the interquartile range, and filled circles indicate outliers.

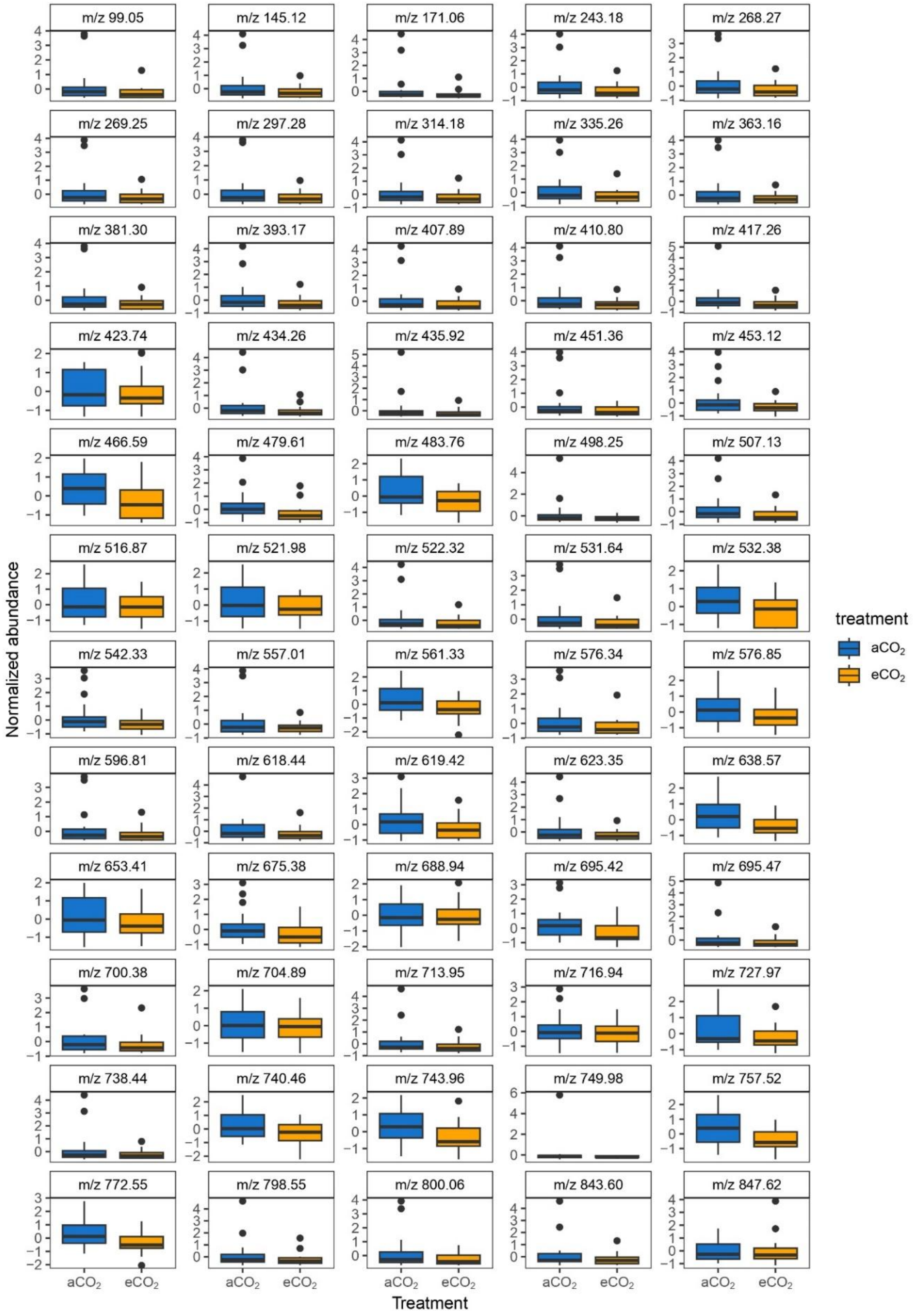

Figure S11d: Unique features (to 2 d.p.) present in root exudates under negative ionisation that are significantly (Kruskal-Wallis, $p<0.05$) depleted under eCO_2. Boxes denote the 25th and 75th percentiles and median lines are given for $n=18$ pseudo-replicates for each treatment (true replication $n=3$), whiskers indicate values up to 1.5x the interquartile range, and filled circles indicate outliers.

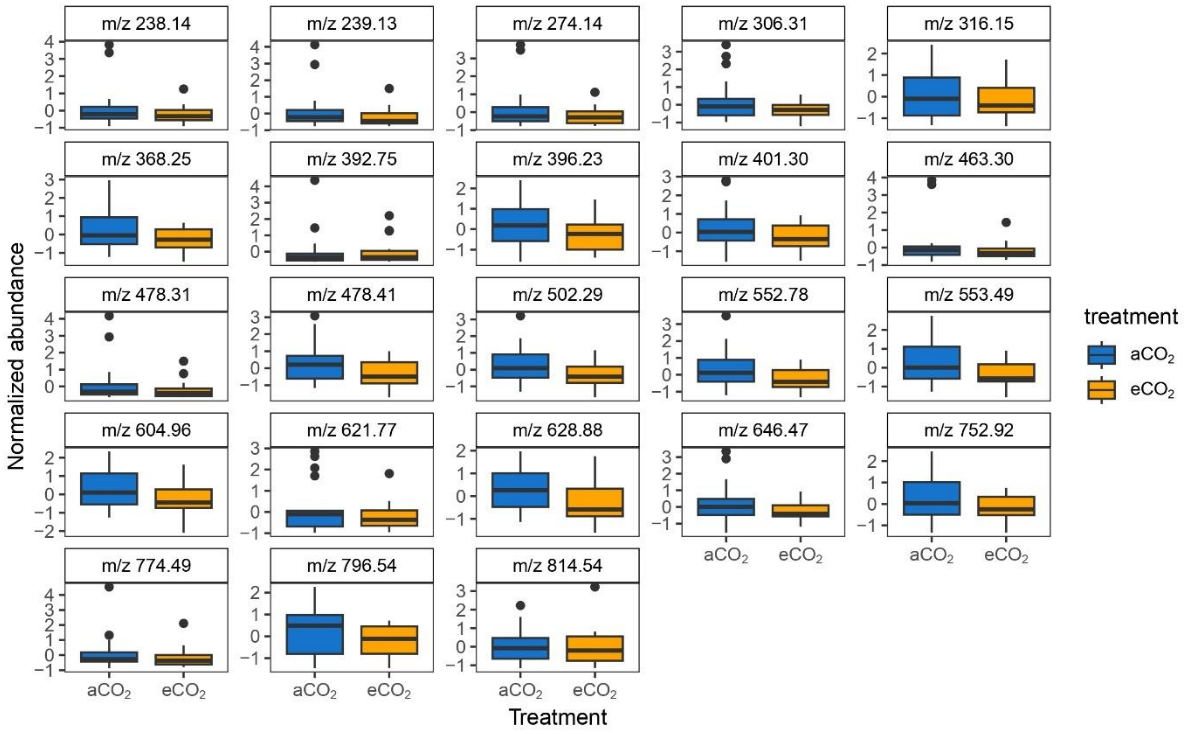

Figure S12a: Unique features (to 2 d.p.) present in roots used for exudate collection under positive ionisation that are significantly (Kruskal-Wallis, *p*<0.05) accumulated under eCO_2. Boxes denote the 25th and 75th percentiles and median lines are given for *n*=18 pseudo-replicates for each treatment (true replication *n*=3), whiskers indicate values up to 1.5x the interquartile range, and filled circles indicate outliers.

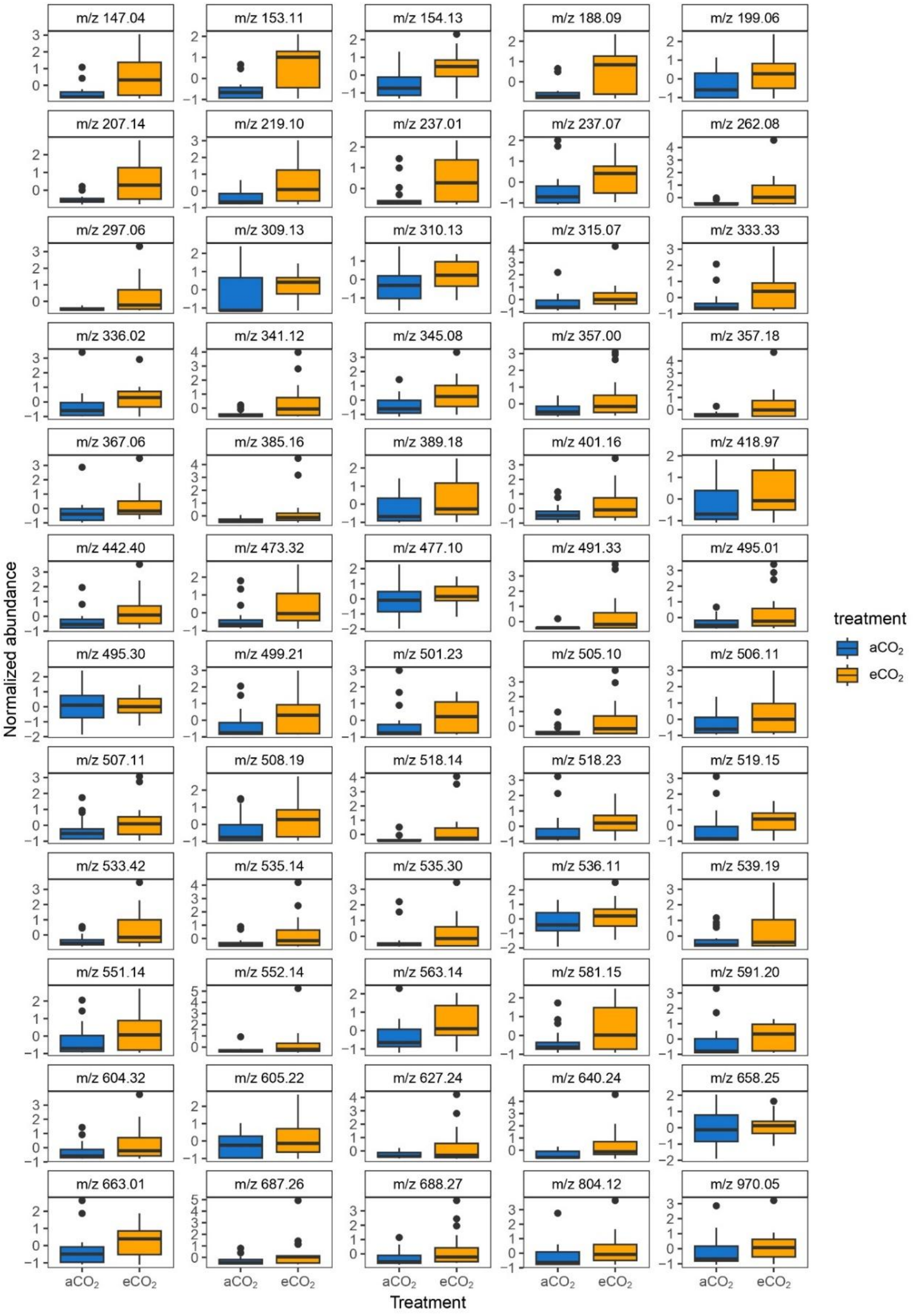

Figure S12b: Unique features (to 2 d.p.) present in roots used for exudate collection under positive ionisation that are significantly (Kruskal-Wallis, $p<0.05$) accumulated under eCO_2. Boxes denote the 25th and 75th percentiles and median lines are given for n=18 pseudo-replicates for each treatment (true replication n=3), whiskers indicate values up to 1.5x the interquartile range, and filled circles indicate outliers**.**

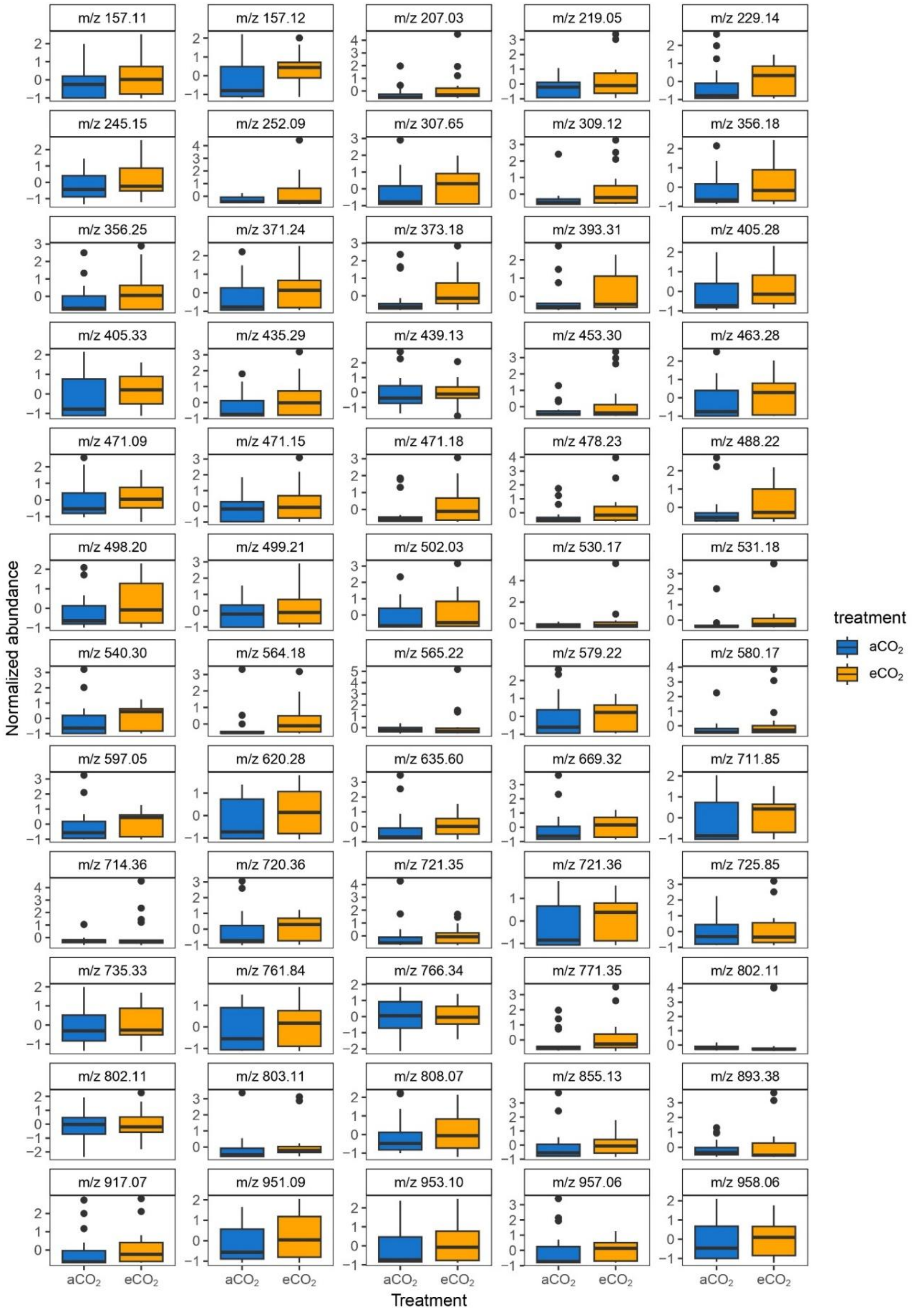

Figure S13a: Unique features (to 2 d.p.) present in roots used for exudate collection under positive ionisation that are significantly (Kruskal-Wallis, p<0.05) depleted under eCO_2. Boxes denote the 25th and 75th percentiles and median lines are given for n=18 pseudo-replicates for each treatment (true replication n=3), whiskers indicate values up to 1.5x the interquartile range, and filled circles indicate outliers**.**

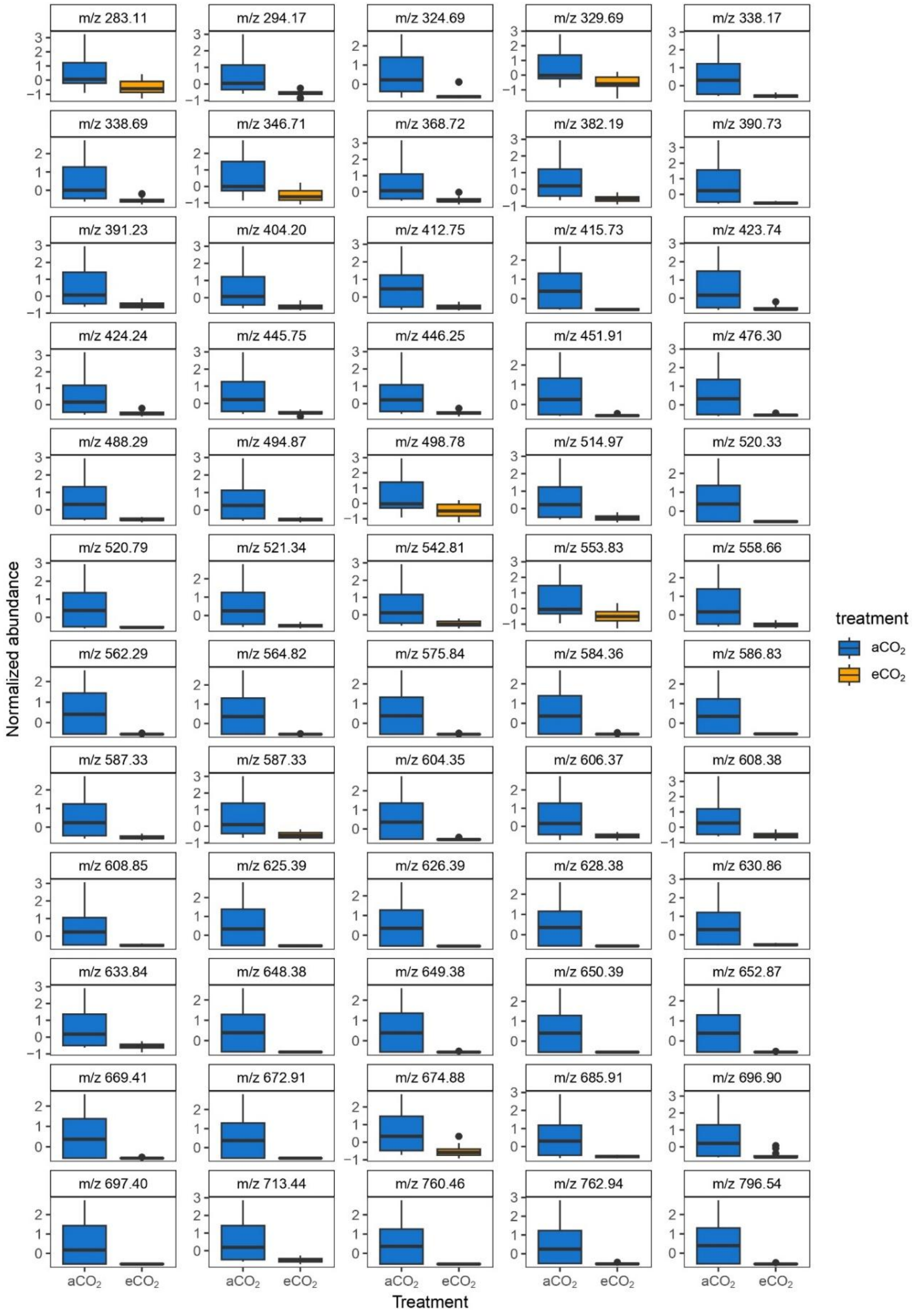

Figure 13b: Unique features (to 2 d.p.) present in roots used for exudate collection under positive ionisation that are significantly (Kruskal-Wallis, $p<0.05$) depleted under eCO_2. Boxes denote the 25th and 75th percentiles and median lines are given for $n=18$ pseudo-replicates for each treatment (true replication $n=3$), whiskers indicate values up to 1.5x the interquartile range, and filled circles indicate outliers**.**

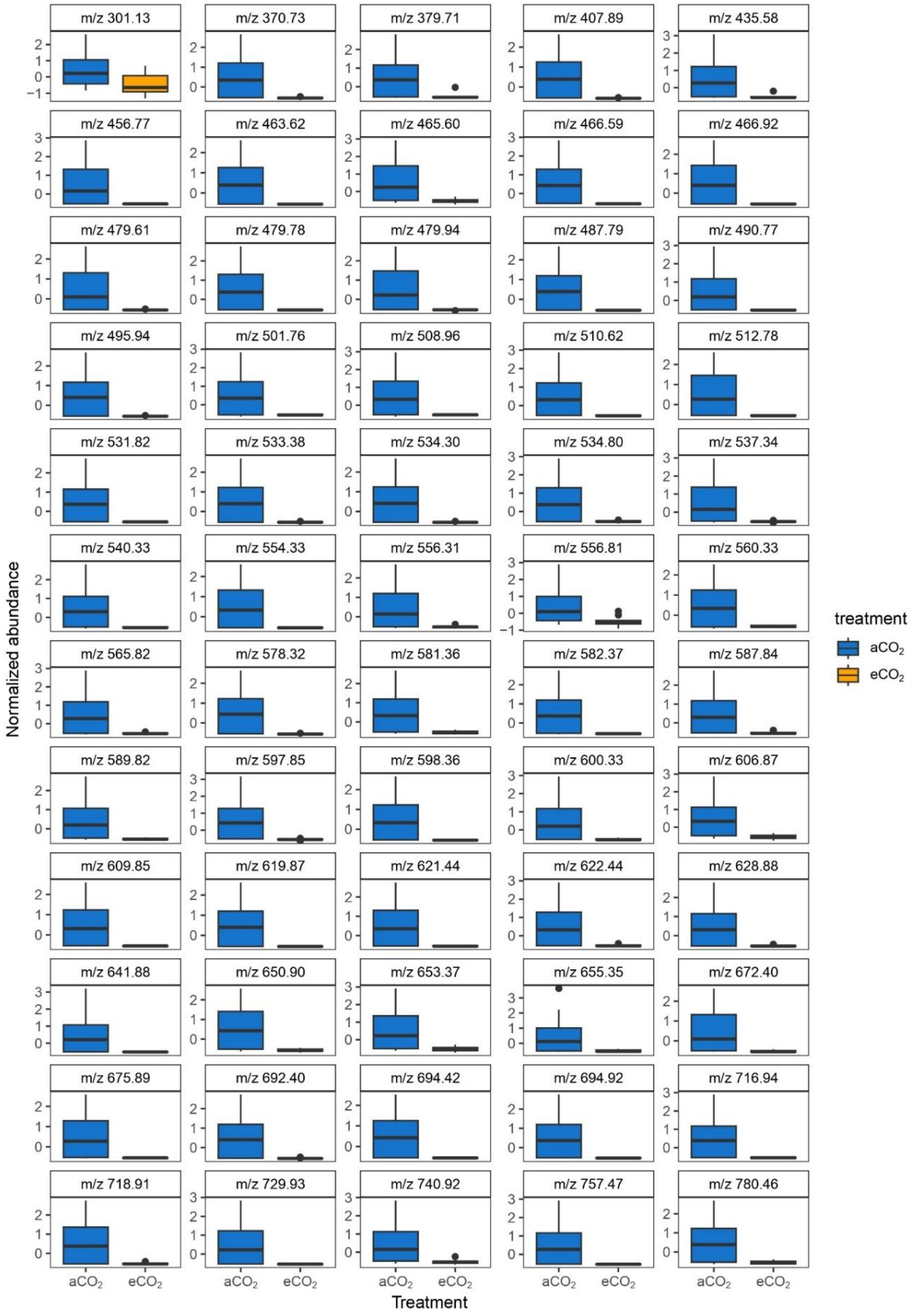

Figure S13c: Unique features (to 2 d.p.) present in roots used for exudate collection under positive ionisation that are significantly (Kruskal-Wallis, $p<0.05$) depleted under eCO_2. Boxes denote the 25th and 75th percentiles and median lines are given for $n=18$ pseudo-replicates for each treatment (true replication $n=3$), whiskers indicate values up to 1.5x the interquartile range, and filled circles indicate outliers**.**

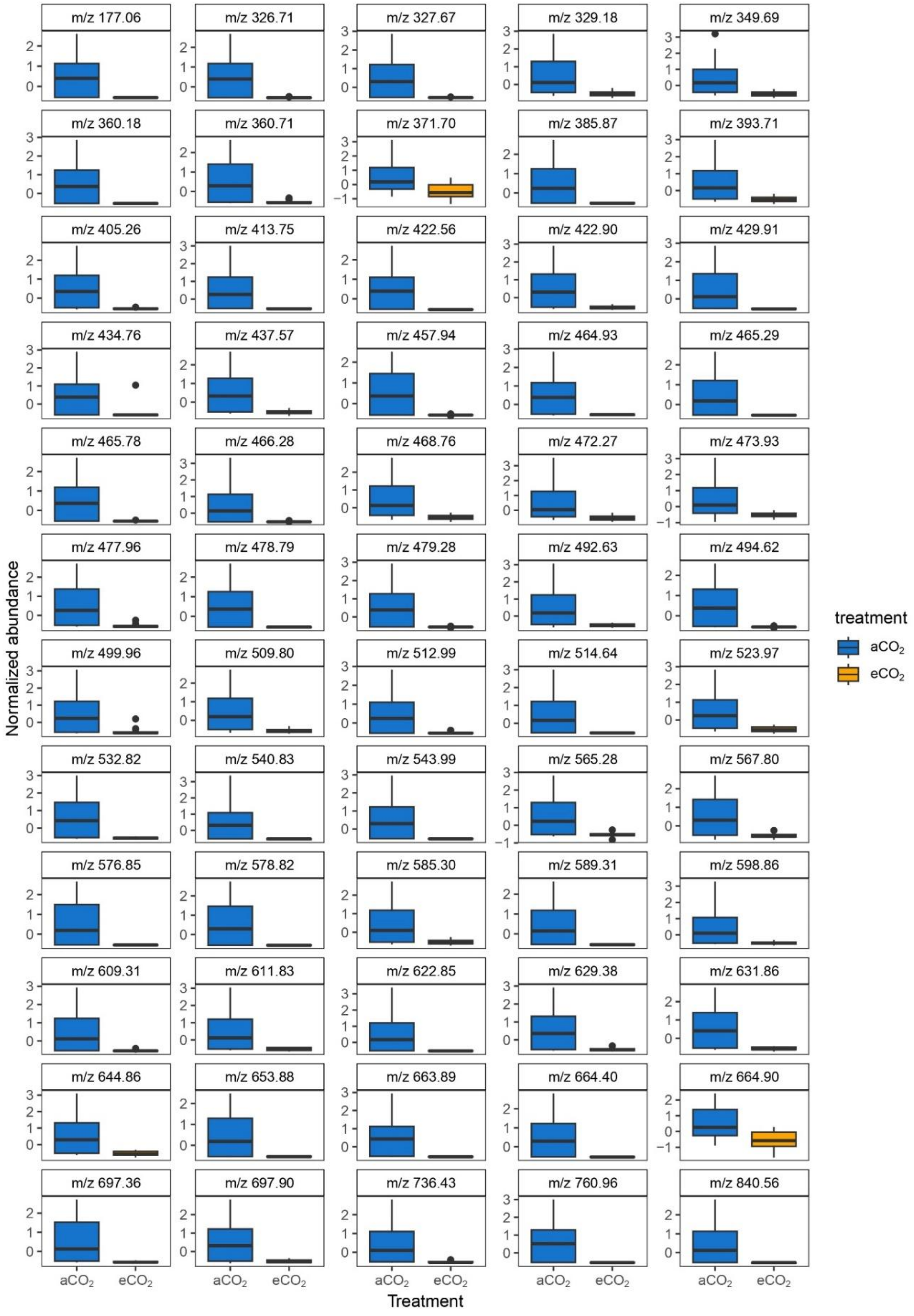

Figure S13d: Unique features (to 2 d.p.) present in roots used for exudate collection under positive ionisation that are significantly (Kruskal-Wallis, p<0.05) depleted under eCO_2. Boxes denote the 25th and 75th percentiles and median lines are given for n=18 pseudo-replicates for each treatment (true replication n=3), whiskers indicate values up to 1.5x the interquartile range, and filled circles indicate outliers.

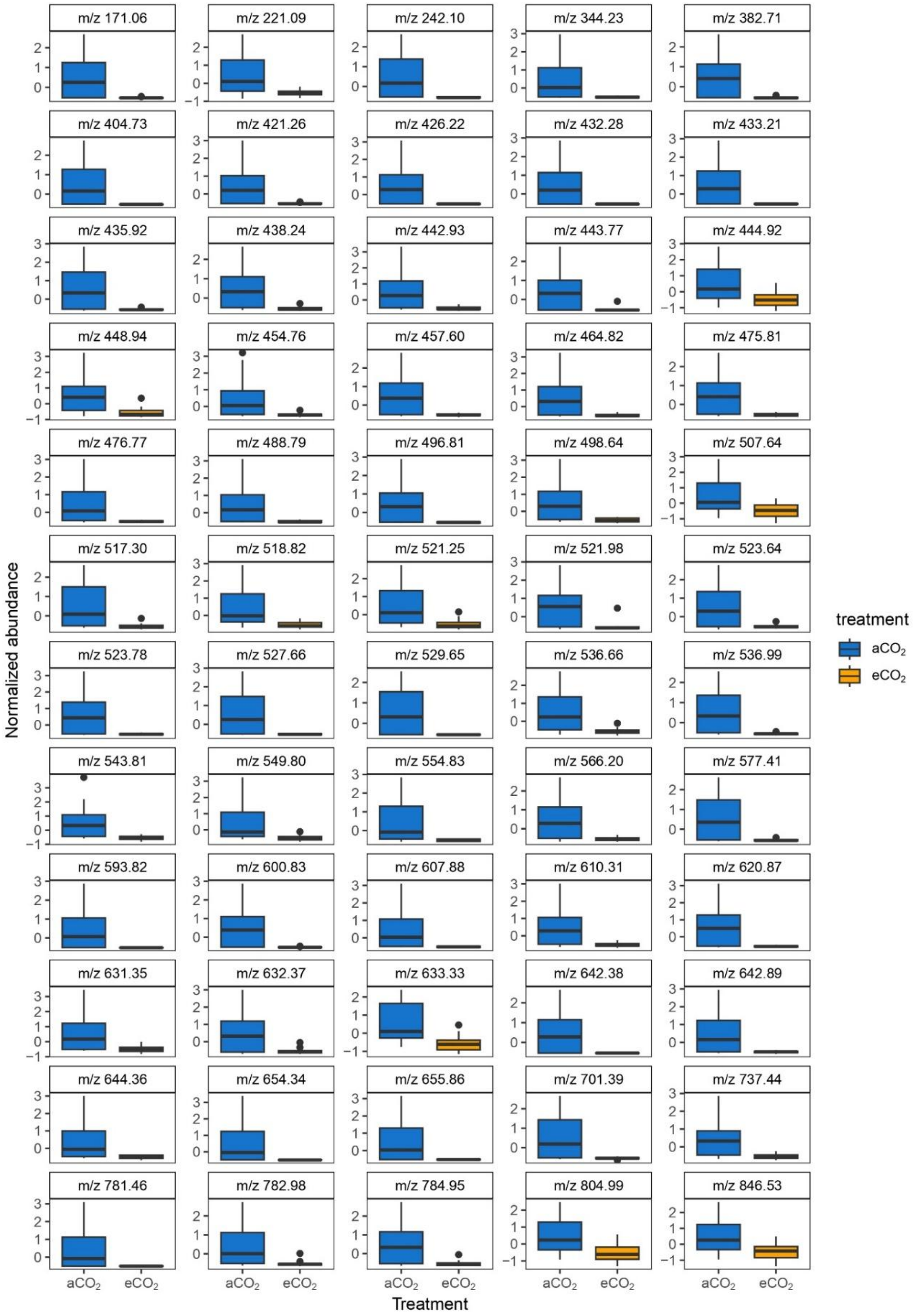

Figure S13e: Unique features (to 2 d.p.) present in roots used for exudate collection under positive ionisation that are significantly (Kruskal-Wallis, $p<0.05$) depleted under eCO_2. Boxes denote the 25th and 75th percentiles and median lines are given for n=18 pseudo-replicates for each treatment (true replication n=3), whiskers indicate values up to 1.5x the interquartile range, and filled circles indicate outliers**.**

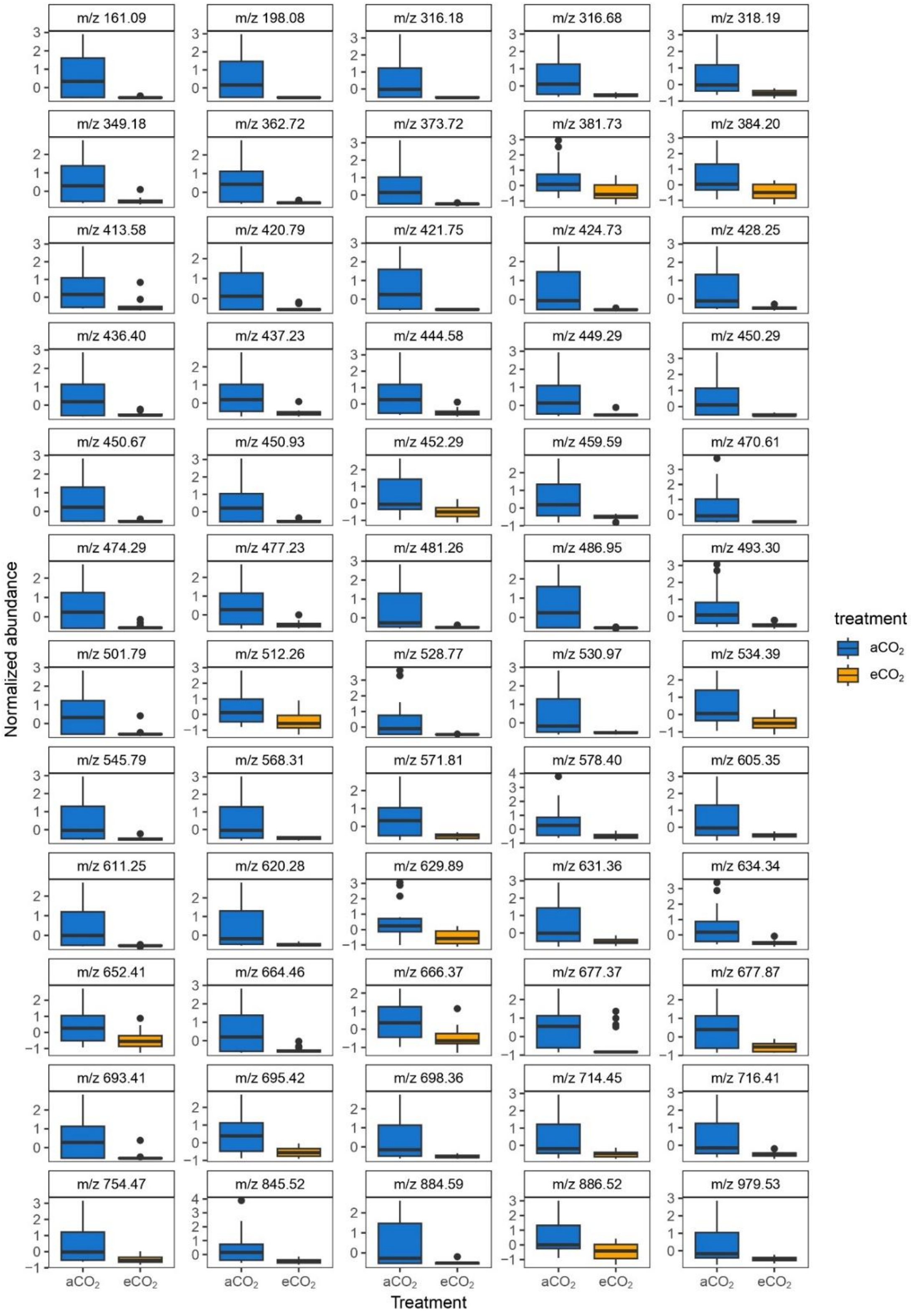

Figure S13f: Unique features (to 2 d.p.) present in roots used for exudate collection under positive ionisation that are significantly (Kruskal-Wallis, p<0.05) depleted under eCO_2. Boxes denote the 25th and 75th percentiles and median lines are given for n=18 pseudo-replicates for each treatment (true replication n=3), whiskers indicate values up to 1.5x the interquartile range, and filled circles indicate outliers**.**

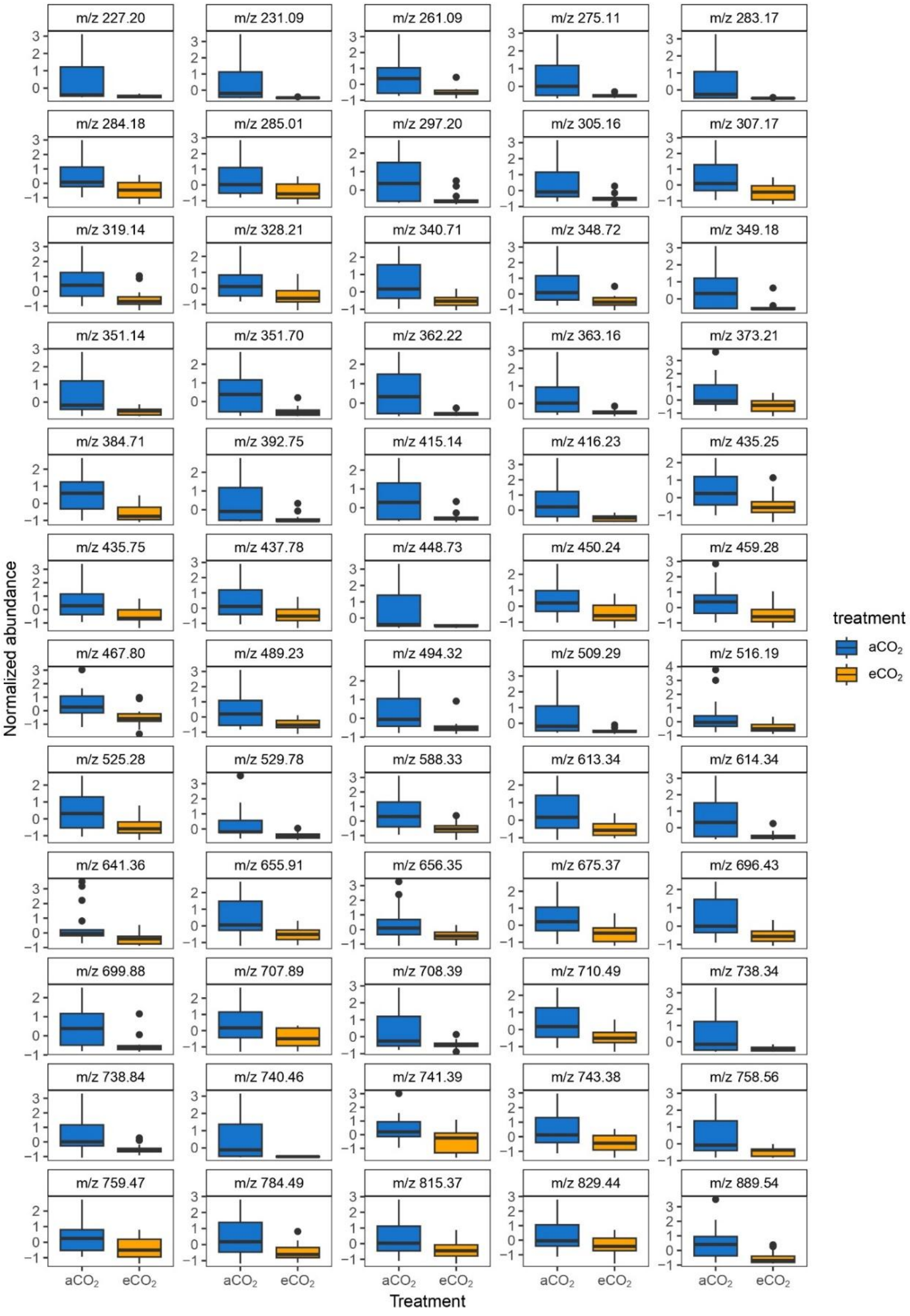

Figure S13g: Unique features (to 2 d.p.) present in roots used for exudate collection under positive ionisation that are significantly (Kruskal-Wallis, p<0.05) depleted under eCO_2. Boxes denote the 25th and 75th percentiles and median lines are given for n=18 pseudo-replicates for each treatment (true replication n=3), whiskers indicate values up to 1.5x the interquartile range, and filled circles indicate outliers**.**

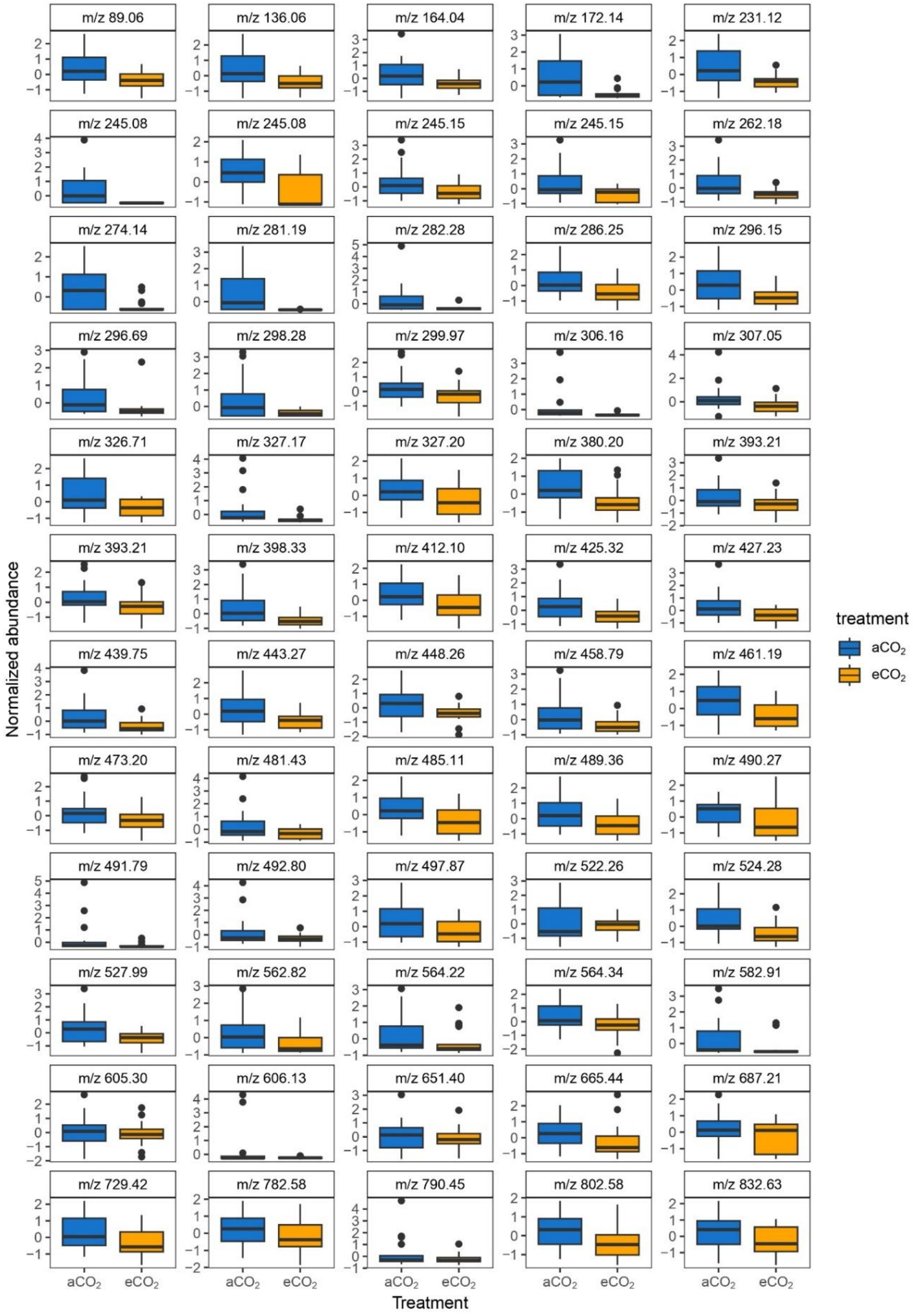

Figure S13h: Unique features (to 2 d.p.) present in roots used for exudate collection under positive ionisation that are significantly (Kruskal-Wallis, $p<0.05$) depleted under eCO_2. Boxes denote the 25th and 75th percentiles and median lines are given for n=18 pseudo-replicates for each treatment (true replication n=3), whiskers indicate values up to 1.5x the interquartile range, and filled circles indicate outliers**.**

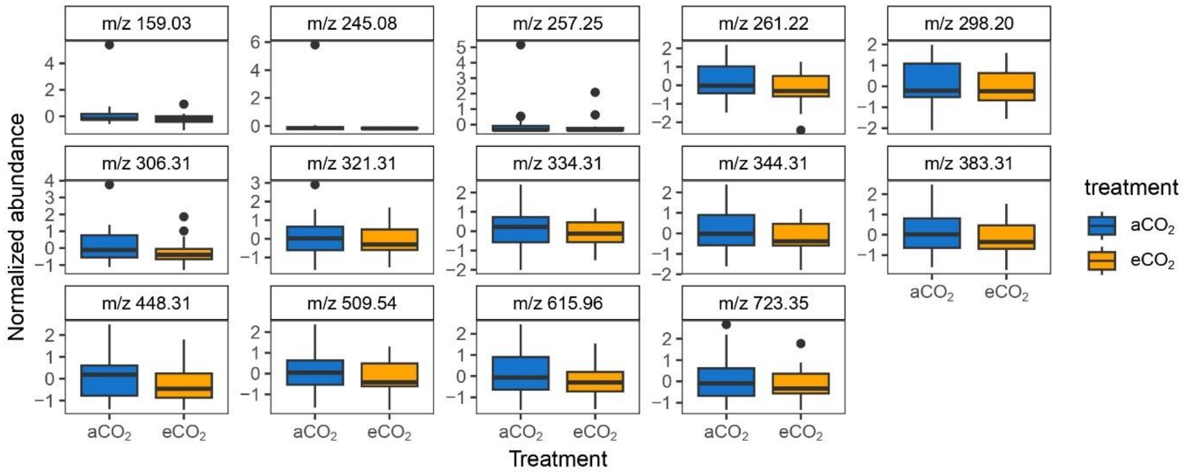

Figure S14a: Unique features (to 2 d.p.) present in roots used for exudate collection under negative ionisation that are significantly (Kruskal-Wallis, p<0.05) accumulated under eCO_2. Boxes denote the 25th and 75th percentiles and median lines are given for n=18 pseudo-replicates for each treatment (true replication n=3), whiskers indicate values up to 1.5x the interquartile range, and filled circles indicate outliers**.**

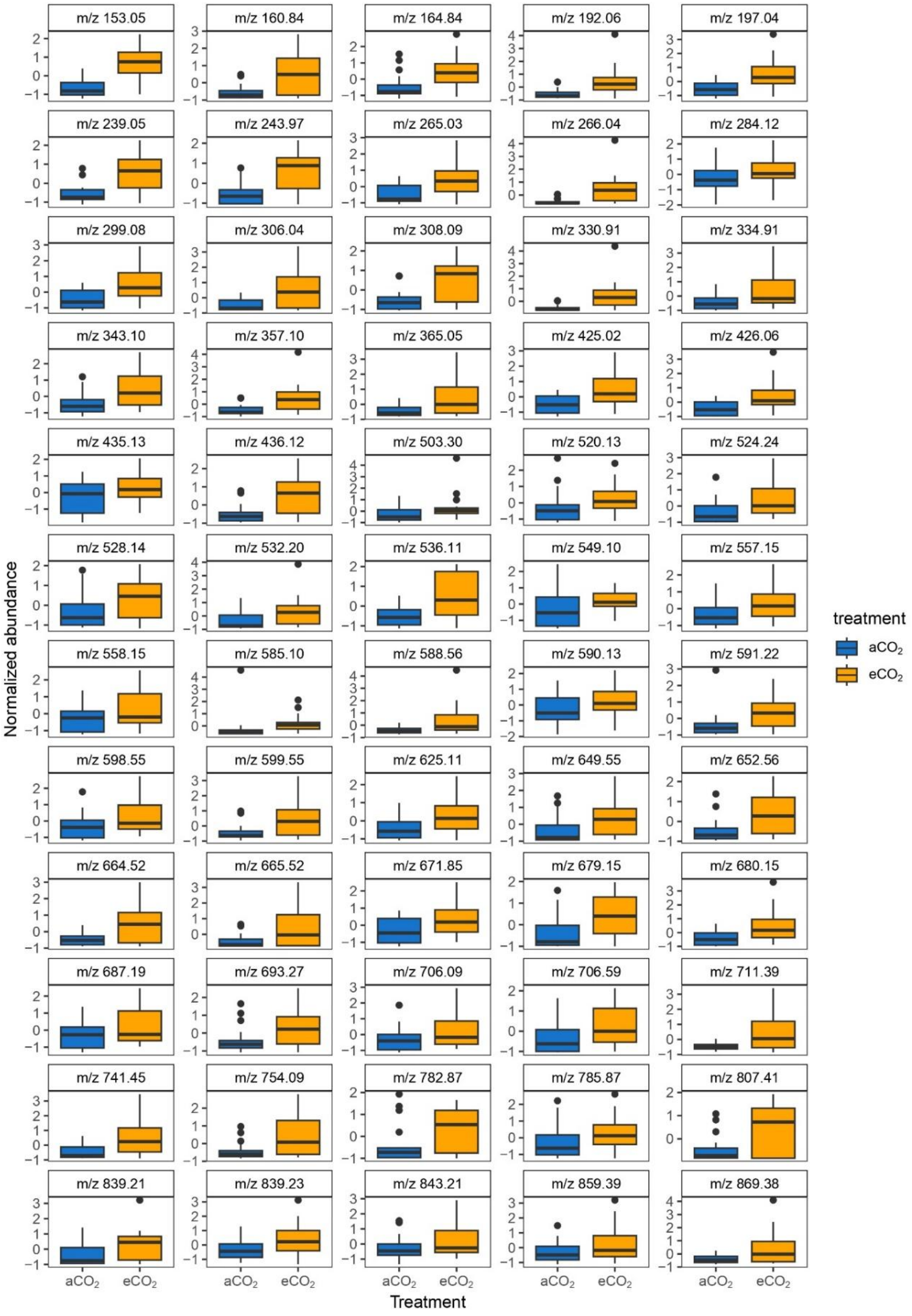

Figure S14b: Unique features (to 2 d.p.) present in roots used for exudate collection under negative ionisation that are significantly (Kruskal-Wallis, $p<0.05$) accumulated under eCO_2. Boxes denote the 25th and 75th percentiles and median lines are given for $n=18$ pseudo-replicates for each treatment (true replication $n=3$), whiskers indicate values up to 1.5x the interquartile range, and filled circles indicate outliers**.**

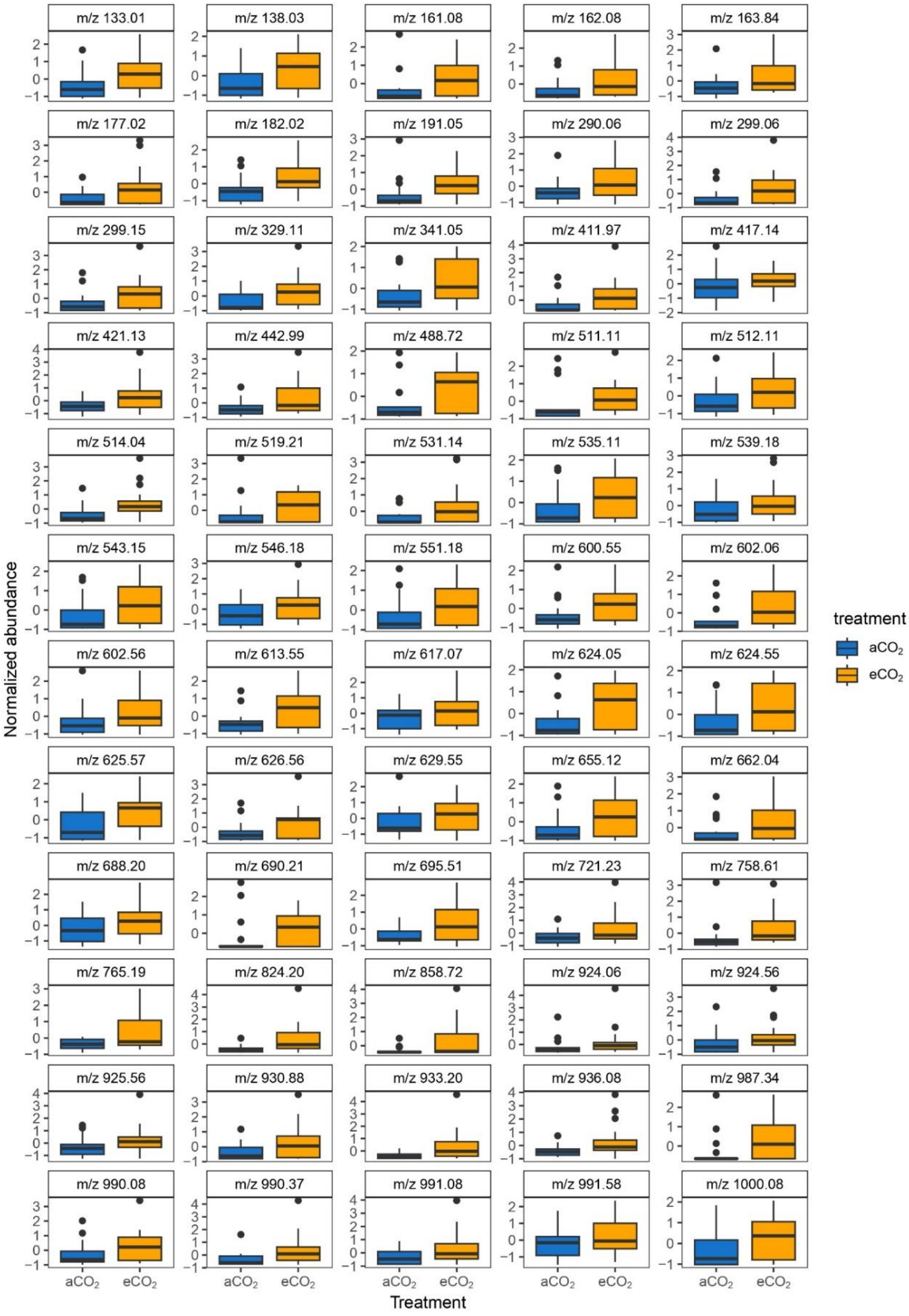

Figure S14c: Unique features (to 2 d.p.) present in roots used for exudate collection under negative ionisation that are significantly (Kruskal-Wallis, $p<0.05$) accumulated under eCO_2. Boxes denote the 25th and 75th percentiles and median lines are given for n=18 pseudo-replicates for each treatment (true replication n=3), whiskers indicate values up to 1.5x the interquartile range, and filled circles indicate outliers**.**

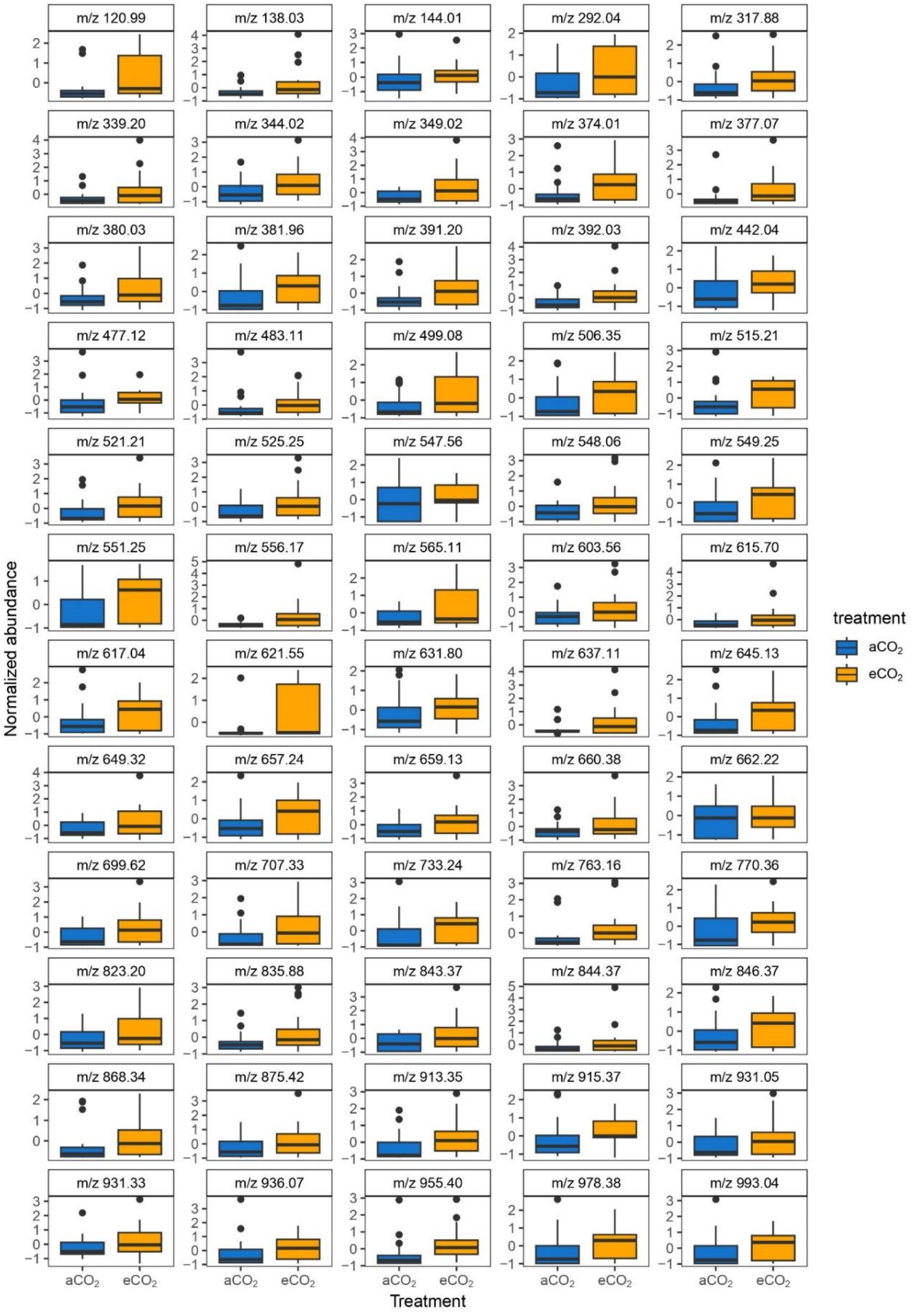

Figure S14d: Unique features (to 2 d.p.) present in roots used for exudate collection under negative ionisation that are significantly (Kruskal-Wallis, $p<0.05$) accumulated under eCO_2. Boxes denote the 25th and 75th percentiles and median lines are given for n=18 pseudo-replicates for each treatment (true replication n=3), whiskers indicate values up to 1.5x the interquartile range, and filled circles indicate outliers**.**

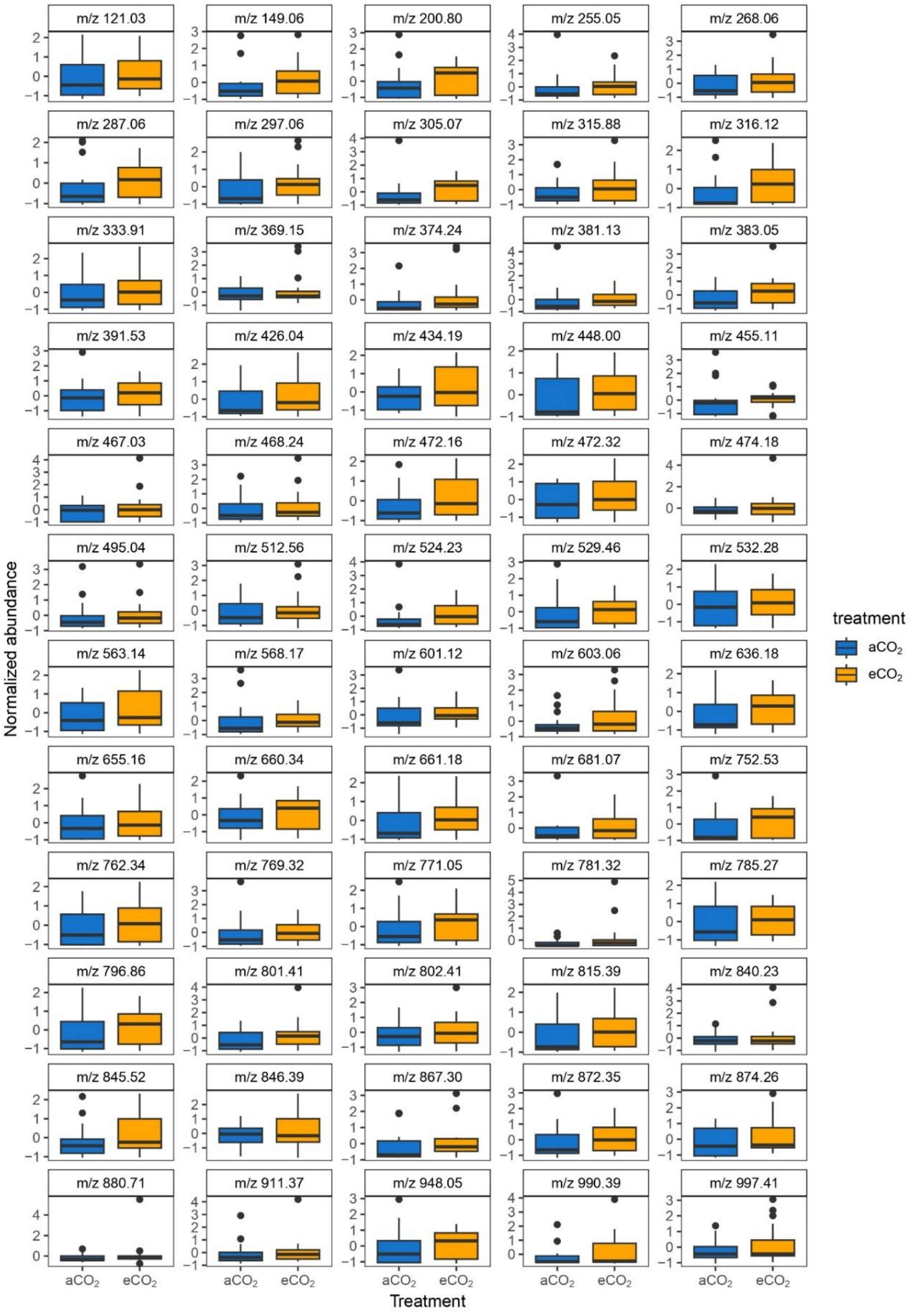

Figure S14e: Unique features (to 2 d.p.) present in roots used for exudate collection under negative ionisation that are significantly (Kruskal-Wallis, p<0.05) accumulated under eCO_2. Boxes denote the 25th and 75th percentiles and median lines are given for n=18 pseudo-replicates for each treatment (true replication n=3), whiskers indicate values up to 1.5x the interquartile range, and filled circles indicate outliers**.**

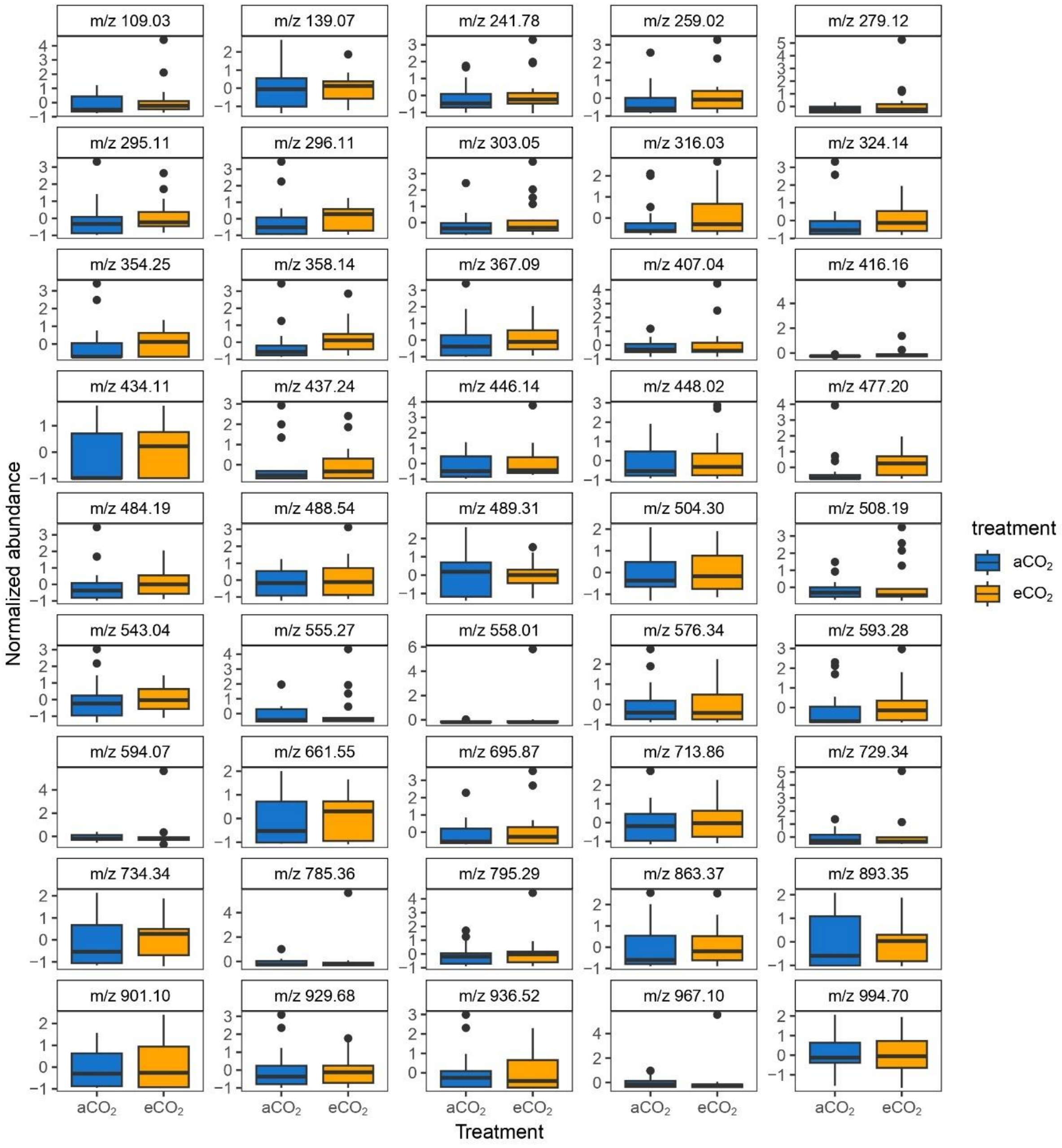

Figure S15a: Unique features (to 2 d.p.) present in roots used for exudate collection under negative ionisation that are significantly (Kruskal-Wallis, $p<0.05$) depleted under eCO_2. Boxes denote the 25th and 75th percentiles and median lines are given for n=18 pseudo-replicates for each treatment (true replication n=3), whiskers indicate values up to 1.5x the interquartile range, and filled circles indicate outliers**.**

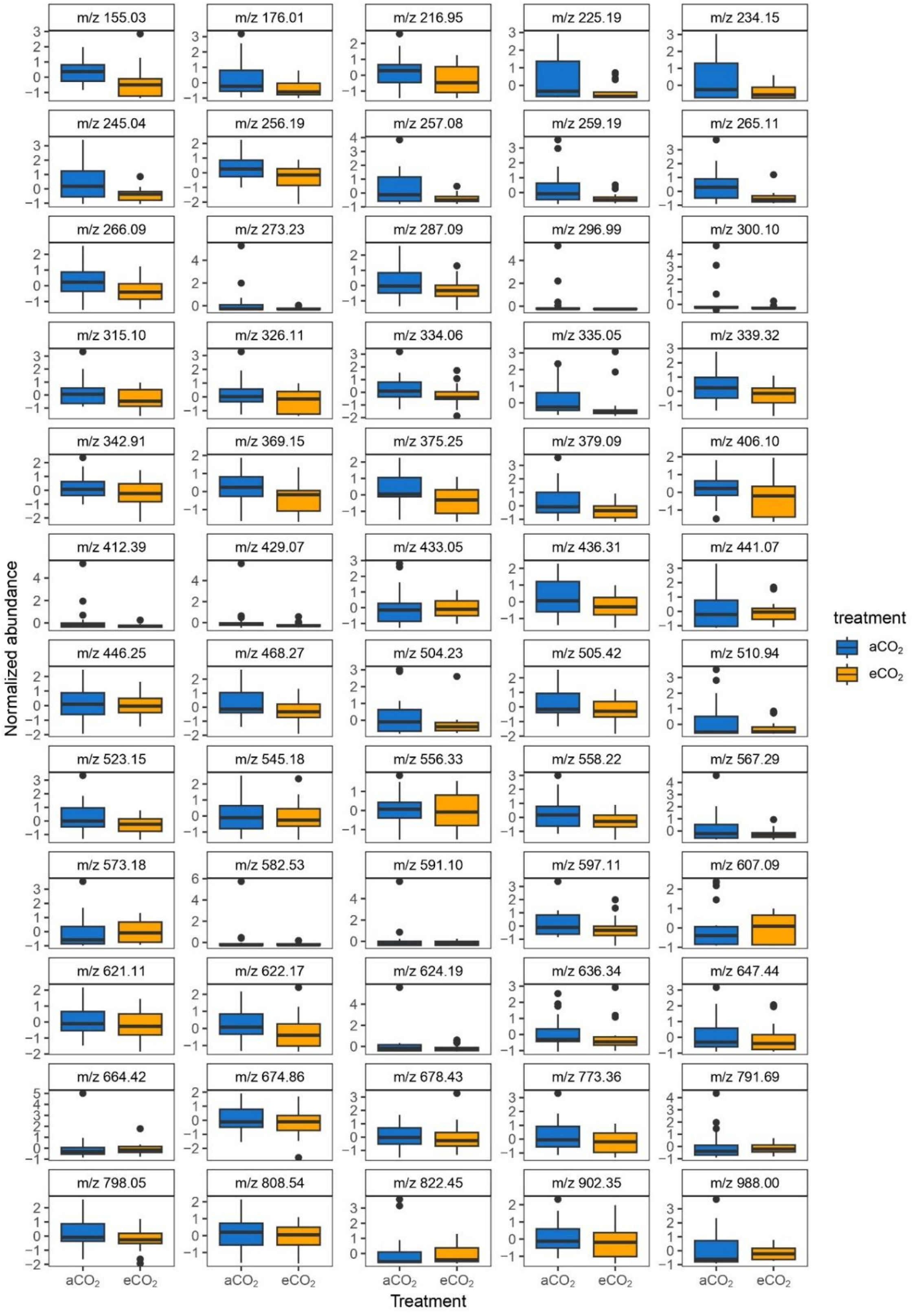

Figure S15b: Unique features (to 2 d.p.) present in roots used for exudate collection under negative ionisation that are significantly (Kruskal-Wallis, $p<0.05$) depleted under eCO_2. Boxes denote the 25th and 75th percentiles and median lines are given for n=18 pseudo-replicates for each treatment (true replication n=3), whiskers indicate values up to 1.5x the interquartile range, and filled circles indicate outliers**.**

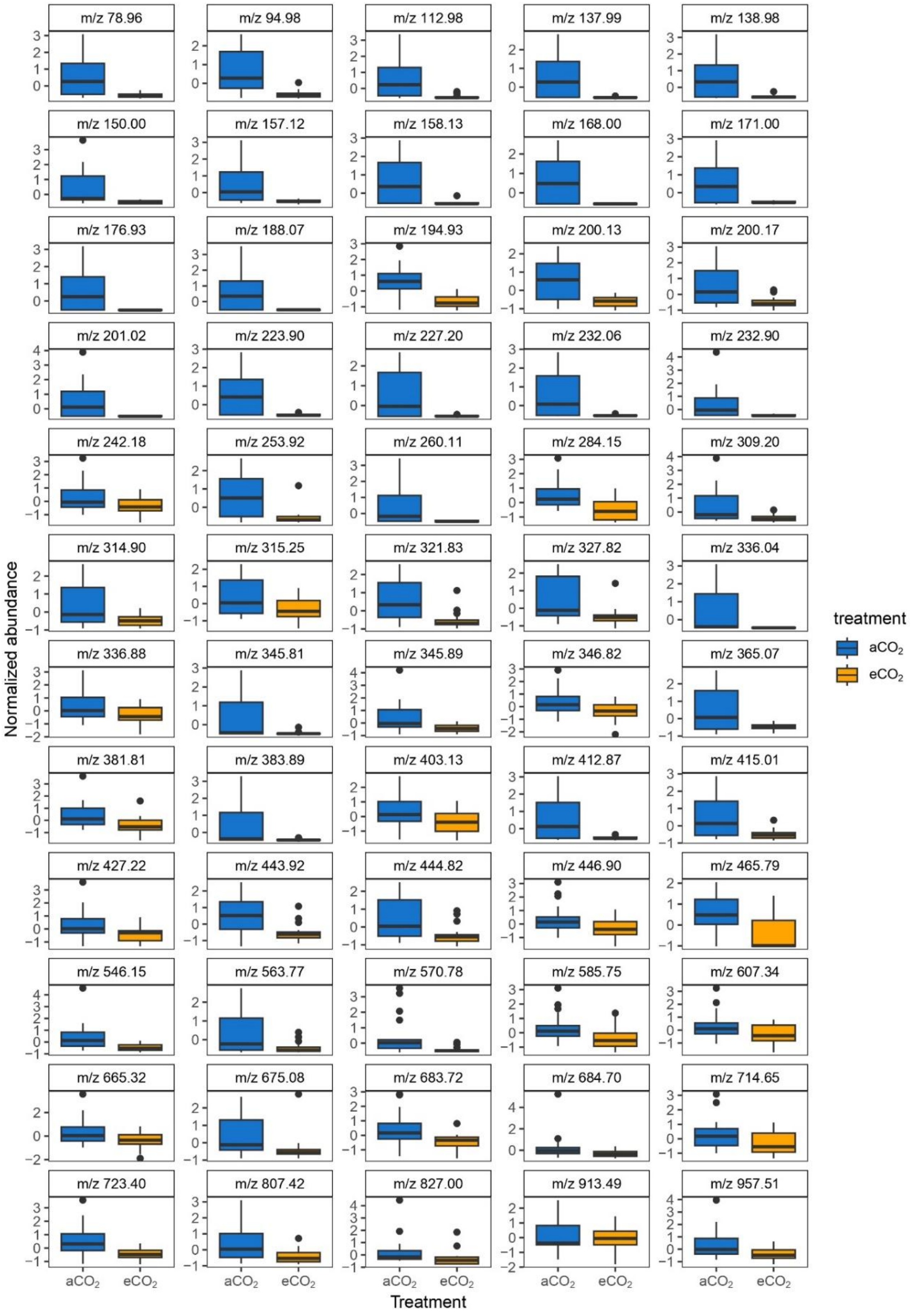

Supplement: Supplementary file 1 — Appendix 01 (PDF) [file pnas.2503595122.sapp.pdf]
